# The Development and Internal Evaluation of a Predictive Model to Identify for Whom Mindfulness-Based Cognitive Therapy Offers Superior Relapse Prevention for Recurrent Depression Versus Maintenance Antidepressant Medication

**DOI:** 10.1177/21677026221076832

**Published:** 2022-04-29

**Authors:** Zachary D. Cohen, Robert J. DeRubeis, Rachel Hayes, Edward R. Watkins, Glyn Lewis, Richard Byng, Sarah Byford, Catherine Crane, Willem Kuyken, Tim Dalgleish, Susanne Schweizer

**Affiliations:** 1 Department of Psychiatry, University of California Los Angeles; 2 Department of Psychology, University of Pennsylvania; 3 National Institute for Health Research (NIHR) Applied Research Collaboration (ARC) South West Peninsula, University of Exeter; 4 Sir Henry Wellcome Mood Disorder Center, University of Exeter; 5 Division of Psychiatry, Faulty of Brain Sciences, University College London; 6 Community Primary Care Research Group, University of Plymouth; 7 National Institute of Health Research Collaboration for Leadership in Applied Health Research and Care, South West Peninsula, England; 8 Health Service and Population Research Department, Institute of Psychiatry, Psychology and Neuroscience, King’s College London; 9 Department of Psychiatry, Medical Sciences Division, University of Oxford; 10 Medical Research Council Cognition and Brain Sciences Unit, University of Cambridge; 11 Cambridgeshire and Peterborough NHS Foundation Trust, Cambridge, England; 12 Department of Psychology, University of Cambridge; 13 School of Psychology, University of New South Wales

**Keywords:** antidepressant medication, depression, mindfulness-based cognitive therapy, precision medicine, relapse prevention

## Abstract

Depression is highly recurrent, even following successful pharmacological and/or psychological intervention. We aimed to develop clinical prediction models to inform adults with recurrent depression choosing between antidepressant medication (ADM) maintenance or switching to mindfulness-based cognitive therapy (MBCT). Using previously published data (*N* = 424), we constructed prognostic models using elastic-net regression that combined demographic, clinical, and psychological factors to predict relapse at 24 months under ADM or MBCT. Only the ADM model (discrimination performance: area under the curve [AUC] = .68) predicted relapse better than baseline depression severity (AUC = .54; one-tailed DeLong’s test: *z* = 2.8, *p* = .003). Individuals with the poorest ADM prognoses who switched to MBCT had better outcomes compared with individuals who maintained ADM (48% vs. 70% relapse, respectively; superior survival times, *z* = −2.7, *p* = .008). For individuals with moderate to good ADM prognoses, both treatments resulted in similar likelihood of relapse. If replicated, the results suggest that predictive modeling can inform clinical decision-making around relapse prevention in recurrent depression.

Globally, depression is now the leading cause of life years lived with disability (Patel et al., 2016, 2018). In many cases, the course of depression is recurrent over the life span (Kessler & Bromet, 2013), even following successful acute-phase interventions (Cuijpers et al., 2013). Successful prevention of the return of depression is therefore key to alleviating the individual and societal burden of depressive disorders. Antidepressant medication (ADM) following successful treatment is currently the predominant preventive intervention targeted at depressive relapse.^1^ Multiple agencies, including the UK National Institute for Health and Care Excellence (NICE), the British Association for Pharmacology (Cleare et al., 2015), and the American Psychiatric Association, recommend both prescription of ADM and mindfulness-based cognitive therapy (MBCT) after remission if a person is deemed at high risk of relapse because of multiple previous episodes or high residual symptoms (Gelenberg et al., 2010; NICE, 2009). An international review of 13 sets of ADM guidelines revealed that recommendations for the duration of such continuation or maintenance^2^ treatment in people deemed at high risk ranged from 1 year to lifelong or indefinite (Piek et al., 2010). Unsurprisingly, therefore, longer term use of ADMs is high and rising (Mojtabai & Olfson, 2014; Organisation for Economic Co-operation and Development, 2013), which accounts for the recorded increase in person-years on ADMs from 0.73 in 1995 to 4.94 in 2012 reported in the United Kingdom (McCrea et al., 2016). The antidepressant benefits of longer term ADM use are tempered by diverse physical and emotional side effects in the majority of patients (Bet et al., 2013; Cartwright et al., 2016), tachyphylaxis and other loss-of-response phenomena (Bosman et al., 2018; Fornaro et al., 2019; Kinrys et al., 2019), and user surveys indicating a desire for evidence-based psychosocial interventions as an alternative to ADMs for all aspects of depression management (Dorow et al., 2018; McHugh et al., 2013; Schweizer et al., 2010).

One such alternative is MBCT, an 8-week, group-based program that has emerged as a leading evidence-based psychological intervention for relapse prevention in recurrent depression (Kuyken et al., 2016). In a multicenter definitive randomized controlled trial (RCT; *N* = 424)—the PREVENT trial (Kuyken, Byford, et al., 2010; Kuyken et al., 2014)—we evaluated MBCT combined with support to taper or discontinue ADM against maintenance of a clinical dose of ADM for 2 years in patients (age ≥ 18 years) with recurrent depression (at least three previous episodes) who were in partial or full remission on ADM.^3^ The trial showed no significant differences in relapse over 2 years between the MBCT and ADM groups (hazard ratio [HR] = 0.89, 95% confidence interval [CI] = [0.67, 1.18]; *p* = .43; relapse rate: 44% MBCT vs. 47% ADM; Kuyken et al., 2015a), a finding corroborated by an individual patient data meta-analysis of 1,258 patients from nine RCTs (Kuyken et al., 2016). Recent meta-analytic work has confirmed and expanded these findings: A network meta-analysis by McCartney et al. (2021) provided additional evidence that MBCT is superior to control conditions in terms of rate of relapse (MBCT vs. treatment as usual) or time to relapse (MBCT vs. treatment as usual or placebo), and a meta-analysis by Breedvelt and colleagues (2021) added evidence of the superiority of combination psychological prevention and continuation ADM over continuation ADM alone.

Given the association between depressive relapse and negative long-term outcomes, helping individuals select the optimal intervention for relapse prevention (from among the available options) is of high importance. Treatment guidelines stipulate that patient preferences should inform treatment selection through a process of shared decision-making (Weston, 2001), and there is some evidence that treatment outcomes are superior for preferred versus nonpreferred treatments (Kwan et al., 2010; Shay & Lafata, 2015; Windle et al., 2019). A critical component of effective shared decision-making is ensuring that comparative evidence for different interventions in the context of the patient’s own clinical profile—what works for whom—is available at the point of care delivery (Winston et al., 2018). This information can come in a variety of different forms, including decision aids (Stacey et al., 2017) or more quantitative outcomes from clinical-prediction models (Bonnett et al., 2019). Recent methodological and empirical advances in “precision medicine” (F. S. Collins & Varmus, 2015) have generated prediction models that provide indices to identify which patients might expect improved clinical outcomes following different acute treatments for depression (Chekroud et al., 2021; Cohen & DeRubeis, 2018; Cohen et al., 2021; Perlis, 2013). A variety of factors are known to predict risk of depressive relapse (Buckman et al., 2018), but clinical prediction models in this area are lacking. Moriarty and colleagues’ (2021) systematic review of prognostic models for predicting depressive relapse identified 10 unique prognostic models, but the studies’ high bias and models’ poor predictive performance suggest that further work is needed.

The participants in PREVENT were assessed at trial baseline on a broad range of psychosocial variables that putatively have a bearing on treatment outcome (Kuyken et al., 2015a). Here, we focus on identifying patient characteristics and constructing prognostic models that could putatively guide the treatment choice between continuing ADM versus MBCT with support to taper or discontinue antidepressant treatment for the prevention of depressive relapse.

## Method

For a checklist corresponding to the TRIPOD (transparent reporting of a multivariable prediction model for individual prognosis or diagnosis) guidelines (G. S. Collins et al., 2015), see the Supplemental Material available online.

### Data set description

The full PREVENT sample comprised 424 individuals randomly assigned (1:1) to ADM or MBCT. Participants with more than 20% missing data on predictor variables (*n* = 15), no data beyond baseline (*n* = 17), or not in receipt of a dose of MBCT deemed sufficient (at least four sessions; following the PREVENT trial protocol: Kuyken et al., 2010, 2015a) for evaluation of MBCT as an intervention alternative (*n* = 25) were excluded from the primary analyses. This led to a sample of 367 participants for the primary analyses. For the data exclusion pipeline, see Figure S1 in SM1 in the Supplemental Material. Sensitivity analyses were performed to probe the impact of removing the 25 who were excluded because of inadequate MBCT dose. The results for this larger sample (*n* = 392) are included in SM2 in the Supplemental Material. Descriptive data for the predictor variables at baseline are provided in the SM3 in the Supplemental Material, as are comparisons of the two treatment groups (ADM vs. MBCT; see Table S1 in the Supplemental Material) and the excluded versus included samples (see Table S2 in the Supplemental Material). These comparisons indicated that there was a significantly greater proportion of women in the ADM group and that ADM participants reported more comorbid diagnoses, had a lower probability that their most recent episode of depression was chronic (≥ 24 months in duration), and were younger, at baseline, compared with the MBCT group (see Table S1 in the Supplemental Material). Relative to the analysis sample, excluded participants were, on average, 4 years younger, had 0.3 more comorbid diagnoses, and reported lower scores on the Dispositional Positive Emotions Scale Curiosity subscale, Self-Compassion Scale Isolation subscale, and the Five-Facets Mindfulness Questionnaire Describe subscale (see Table S2 in the Supplemental Material).

### Predictor variables

The PREVENT study included a wide range of 53 potential demographic, clinical, and psychological predictor variables (Table 1). The demographic and clinical predictors were selected because they are available in clinical practice, and indeed, many are commonly included as part of routine diagnostic procedures. Psychological predictors included standardized self-report measures of potential mechanisms of treatment efficacy (including mindfulness, self- and other-compassion, and repetitive thinking).

**Table 1.** Predictors Included in the Variable-Selection Analysis

Variable	Description
Demographic	
Age	Age at baseline in years
Gender	Binary variable, reflecting self-identified gender: female or male (variable was made dichotomous because most individuals identified as one or other gender)
Education	Level of education attained, in which 0 = no educational qualification, 1 = O levels or GCSEs, 2 = AS and A levels (UK Advanced Level), 3 = vocational training/qualification, 4 = university bachelor’s degree, 5 = university master’s degree, and 6 = university professional training/PhD
Relationship status	Binary variable: no (single/divorced/widowed) versus yes (married/civil partnership/cohabiting)
Employment status	Binary variable: unemployed versus full- or part-time
Clinical	
Clinician-rated depressive symptoms	The total score on the GRID-Hamilton Rating Scale for Depression (GRID-HAMD; Williams et al., 2008) was used as an index of clinician-rated depressive symptoms. The GRID-HAMD is a scale that offers explicit standardized scoring guideline for the clinician-rated assessment of depression. The scale consists of 17 items assessing symptoms of depression that are rated on a scale from 0 (*not present*) to 4 (*severe*).
Self-reported depressive symptoms	The total score of the Beck Depression Inventory–II (Beck et al., 1996) was used to assess self-reported symptoms of depression. The 21-item scale requires participants to endorse symptom levels ranging from 0 (*not present*) to 3 (*severe*).
Age of depression onset	Age at first depressive episode
Chronicity	Binary variable indicating whether the duration was ≥ 24 months
Previous psychological treatment	Binary variable indicating whether the participant had received a previous psychological treatment
Previous suicide attempt	Binary variable indicating whether the participant had previously attempted suicide or not
Family history of depression	Binary variable indicating whether the participant reported a family history of mood disorders or not
Comorbidity	Number of comorbid diagnoses
Psychological	
Validated questionnaires^a^	
Five Facet Mindfulness Questionnaire (FFMQ; Baer et al., 2006)	The FFMQ measures five facets of mindfulness: (a) Observe – observing internal and external experiences (eight items); (b) Describe – describing internal experiences/states verbally (eight items); (c) Aware – acting with awareness (eight items), (d) Non-Judging – a nonjudgmental stance toward one’s thoughts and feelings (eight items), and (e) Non-Reactivity – allowing thoughts and feelings to come and go (seven items). Individuals rated the extent to which they experienced these states ranging from 1 (*never or very rarely true*) to 5 (*very often or always true*).
Self-Compassion Scale (SCS; Neff, 2003)	The SCS consists of six self-compassion subscale factors: Self-Kindness (five items), Self-Judgment (five items), Common Humanity (four items), Isolation (four items), Mindfulness (four items), and Over-Identification (four items). We additionally included a bespoke subscale that assesses compassion for others. Ratings are provided on a scale ranging from 1 (*almost never*) to 5 (*almost always*).
Dispositional Positive Emotion Scale (DPES; Shiota et al., 2006)	We included the following DPES subscales: Joy (six items), Contentment (five items), Love (six items), Compassion (five items), and Awe (six items). We additionally included a bespoke subscale that assesses Curiosity for internal and external experiences. Ratings were provided ranging from 1 (*strongly disagree*) to 5 (*strongly agree*).
Cognitive Emotion Regulation Questionnaire (CERQ; Garnefski et al., 2001)	The CERQ is a 36-item questionnaire assessing individuals’ propensity to employ four maladaptive (Catastrophizing, Rumination, Other-Blame, and Self-Blame) and five adaptive (Acceptance, Positive Refocusing, Positive Reappraisal, Putting into Perspective, and Refocus on Planning) emotion-regulation strategies when they were confronted with negative events. Item ratings ranged from 1 (*almost never*) to 5 (*almost always*).
Cambridge-Exeter Repetitive Thought Scale (CERTS; Barnard et al., 2007)	The CERTS assesses individuals’ dispositional tendency for Brooding (Section 1), the temporal course of their brooding thinking (Section 2), dispositional tendency for repetitive thinking in general (Section 3), difficulties disengaging from repetitive thinking (Section 4), and attitudes toward repetitive thinking (Section 5). For Sections 1 through 4, responses were provided with respect to eight scenarios: (a) feeling sad, (b) feeling happy, (c) feeling angry, (d) feeling anxious, (e) being with others, (f) being alone, (g) experiencing a set-back, and (h) making progress. In Sections 1, three to five items were given ratings ranging from 1 (*almost never*) to 5 (*almost always*), and in Section 2, items were rated from *only moments* to *what seems like hours*.
Measure of Parental Style (MOPS; Parker et al., 1997)	The MOPS was administered to assess levels of parental abuse experienced as a child. Participants indicate to what extent 15 statements about their mother and father (30 items total) were true for the first 16 years of their lives. Participants rated the statements from 0 (*not at all true*) to 3 (*extremely true*). A median split was used to categorize participants as high or low (see Kuyken et al., 2015).
General Self-Efficacy Scale (GSE; Schwarzer & Jerusalem, 1995)	The GSE is a 10-item scale that assessed individuals’ sense of self-efficacy over the past 2-week period. Participants answered the scale on items from 1 (*definitely disagree*) to 5 (*definitely agree*).
Bespoke measures	
Stigmatization and normalization (SN)	SN was a bespoke seven-item questionnaire asking individuals to indicate how often they experienced stigmatization because of their depression. Items were rated on a scale from 1 (*almost never*) to 5 (*almost always*).
Warning signs (WS)	WS was a bespoke six-item questionnaire assessing individuals’ ability to recognize warning signs of depression. Responses ranged from 1 (*almost never*) to 5 (*almost always*).
Relationship satisfaction (RS)	RS was assessed with a bespoke questionnaire that individuals were asked to complete thinking of the most important relationship in their lives. The scale’s seven items assess relationship satisfaction on a scale ranging from 1 (*almost never*) to 5 (*almost always*).
Preference for mindfulness- based cognitive therapy (MBCT)	Item assessing participants’ sentiment about being assigned to MBCT (Question: “How do you feel about the possibility of being in an MBCT group”), rated on a Likert scale from 1 (*not positive at all*) to 5 (*extremely positive*).
Preference for antidepressant medication (ADM)	Item assessing participants’ sentiment about being assigned to ADM (Question: “How do you feel about remaining on your ADMs”), rated on a Likert scale from 1 (*not positive at all*) to 5 (*extremely positive*).
Preference for therapy type	Item assessing participants’ preferred treatment option (Question: “Do you have a preference for a group”), rated on a Likert scale from 1 to 5 (1 = *MBCT*, 3 = *no preference*, 5 = *continue on ADM*).

Note: Individuals were asked to complete all measures with respect to the previous 2 weeks. The scaling was standardized to facilitate interpretation from factor analyses and similar computations planned for the trial. The labels of the original scales were maintained. GCSE = General Certificate of Secondary Education.

a These scales were scored on a 5-point Likert scale irrespective of their original scoring range.

Missing predictor variable data at baseline were imputed using the full (*N* = 424) sample via the *missForest* (Version 1.4; Stekhoven & Bühlmann, 2012) package for the R software environment (Version 3.5.1; R Core Team, 2018), which implements a random-forest-based nonparametric imputation approach. Random-forest-based imputation compared favorably in several evaluations of different imputation approaches (Hong & Lynn, 2020; Shah et al., 2014; Stekhoven & Bühlmann, 2012; Waljee et al., 2013).

For the 53 potential predictors assessed at baseline in the PREVENT data, following imputation, continuous variables were *z*-scored, and dichotomous variables were set to −0.5 and 0.5. No outcome data were included in the imputation of the missing baseline data. Note that the education variable was imputed as an ordered categorical variable and then was converted into a continuous (numeric) variable for the remainder of the analyses.

### Statistical approach to treatment selection

An in-depth discussion of how data can be used to create and evaluate treatment recommendations can be found in a recent review of treatment selection (Cohen & DeRubeis, 2018). The core concept is that statistical models are constructed and used to generate predictions for an individual in two (or more) treatments, and then those predictions are used to determine which treatment to recommend (Cohen et al., 2019). Much of the work in this space (e.g., the Personalized Advantage Index approach; DeRubeis et al., 2014) has been based on the proportional-interaction model. Luedtke and colleagues (2019) highlighted potential problems with the use of this approach in the small RCT samples that are often available, including the fact that implicit estimation and testing of interaction effects (vs. main effects) requires larger samples. Their simulation work suggested that sample sizes of at least 300 per condition are required for adequate statistical power to detect clinically significant improvements in response associated with model-based treatment selection. Other approaches that have been demonstrated rely solely on prognostic models (e.g., Lorenzo-Luaces et al., 2017; Wiltsey Stirman et al., 2021). For a discussion and contrasting of these different approaches, see Cohen et al. (2021). Following the approach proposed by Kessler et al. (2017) and demonstrated by Deisenhofer and colleagues (2018), we constructed separate prognostic algorithms for each of our two treatment conditions (MBCT and ADM). For each patient, a “factual prediction”—how well they were expected do in their actual treatment arm on the basis of their scores on the variables selected for that treatment’s prognostic model—was generated, as was a “counterfactual prediction”—how well they would hypothetically have done in the alternative treatment arm on the basis of their scores on the predictors that were included in the prognostic model for the alternative treatment arm.

In this approach, the predictive performance of each of the two separate treatment arm algorithms could be independently evaluated (see below for information about cross-validation) by comparing the factual predictions with the observed outcomes. If both algorithms yielded inaccurate factual predictions, this would have revealed that the data, or the modeling procedures that were implemented, did not provide a useful signal for prediction purposes. If both models yielded accurate factual predictions, the computed difference between the sets of predictions for MBCT and ADM could have served as an index for each patient that indicated which of the two treatments would be optimal (Cohen & DeRubeis, 2018). Finally, if only one of the models (e.g., Tx-A) yielded accurate factual predictions, that model on its own could be evaluated for its potential utility for guiding treatment decisions. Patients could be arrayed according to their predicted outcome in the condition with the reliable prognostic model (Tx-A). In the absence of reliable information about expected response to the other treatment (Tx-B) and the assumption that the two treatments yielded similar outcomes on average, participants with poor prognoses in Tx-A could be reasonably advised to try Tx-B, whereas a sensible recommendation for those with good prognoses in Tx-A would be Tx-A. Thus, the expectation in this scenario would be that differential response would be observed across the spectrum of Tx-A prognoses.

We applied this approach to the PREVENT data, and below we outline the steps of variable selection, cross-validation, and assessment of model fit involved in building and evaluating the prognostic algorithms for MBCT and ADM. Although analyses revealed the MBCT model to have poor predictive performance (as indicated by low area under the curve [AUC]), the ADM model evidenced good predictive performance and was superior to a benchmark model constructed using only baseline depression severity. Consequently, we generated and evaluated the treatment-selection indices using the ADM prognostic model only. This allowed us to ask the question of whether there were differential outcomes for participants who received MBCT versus ADM in patients predicted to do well, moderately, or poorly if they continued with ADM. The details regarding the model building and evaluation for the poorly fitting MBCT model are described in detail in SM4 in the Supplemental Material.

#### Cross-validation

When using cross-validation in the context of predictive model evaluation, it is essential to protect against “double-dipping” (Hastie et al., 2009). For example, it is critical that the predictions that are evaluated are generated from models that are constructed (in terms of variable selection, hyperparameter tuning, and weight setting) without the use of data from individuals for whom the predictions are being made.

**Fig. 1.**
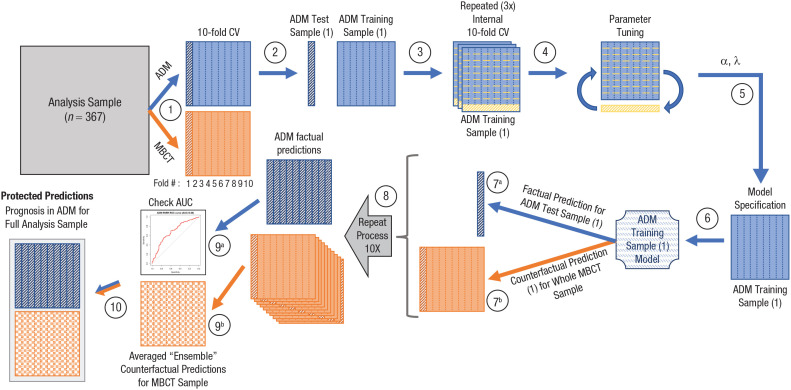
Schematic of cross-validation procedure for producing antidepressant medication (ADM) predictions for the full analysis sample. Ten key steps in the procedure are indicated by circled numbers. Step 1 (10-fold cross-validation [CV]): The main analysis sample was separated into ADM and mindfulness-based cognitive therapy (MBCT) samples, each of which was then split into 10 subgroups, balanced on outcomes. Step 2: The ADM sample was separated into its first train-test samples, and the first of the 10 subgroups was held out as ADM test sample (1); the other nine subgroups constituted ADM training sample (1). Steps 3 and 4: ADM training sample (1) was then itself split into 10 subgroups, and parameter tuning was performed using internal 10-fold CV; this entire process was repeated three times using different random permutations of the internal 10-fold CV of ADM training sample (1). Step 5 (hyperparameter optimization): The optimal alpha (a) and lambda (λ) were selected and used in Step 6 (model specification), in which elastic-net regularized regression (ENRR) was applied to the entire ADM training sample (1) to derive the ADM training sample (1) Model. Step 7^a^: This model was then used to generate factual predictions for the held-out ADM test sample (1) and to generate counterfactual predictions (Step 7^b^) for the entire MBCT sample. Step 8: Steps 2 through 7 were then repeated nine more times to complete the 10-fold CV. Step 9^a^: The resulting set of (protected) factual predictions for the entire ADM sample (likelihood of relapse in ADM) were then evaluated using the area under the receiver operating characteristic curve. Step 9^b^: The set of 10 (protected) counterfactual predictions for each individual in the MBCT sample (likelihood of relapse if they had received ADM) were averaged, which resulted in a set of averaged “ensemble” counterfactual predictions for the MBCT sample. Step 10: The ADM and MBCT samples and their ADM predictions were then recombined, which resulted in protected prognoses under ADM for the full analysis sample.

We performed 10-fold cross-validation (Hastie et al., 2009), which involved splitting both the ADM and MBCT samples into 10 subgroups, balanced on outcomes (Fig. 1, Step 1). Each of the 10 ADM subgroups was then held out (Fig. 1, Step 2), and a prediction model was constructed using the remaining nine ADM subgroups as the training sample (Fig. 1, Steps 3–6). That model was then applied to the 10th ADM group to generate factual predictions of expected response in ADM (Fig. 1, Step 7^a^) and was also applied to the entire MBCT sample to generate counterfactual predictions of their expected response if they had received ADM (Fig. 1, Step 7^b^). The protections needed differ when generating factual and counterfactual prediction for each treatment arm. When predicting ADM outcomes for the MBCT sample, no cross-validation is needed because the ADM model was constructed without the MBCT individuals and thus can be applied to these individuals without concern over double-dipping.

This process was repeated nine more times for each of the other nine ADM subgroups (Fig. 1, Step 8), which resulted in the generation of a single “protected” factual prediction for each of the individuals in the ADM condition. The 10 protected counterfactual predictions (one from each of the 10 ADM models) for each of the individuals in the MBCT condition were averaged to create an ensemble counterfactual prediction of how patients who received MBCT would have been expected to fare had they received ADM (Fig. 1, Step 9^b^). The analogous process was then performed for the MBCT group, which resulted in each individual in MBCT receiving a single factual prediction of their outcomes in MBCT and patients in the ADM condition receiving ensemble counterfactuals for their expected outcomes had they received MBCT (see Fig. S3 in the Supplemental Material).

Finally, to provide a benchmark to help in the evaluation of these multivariable prediction models, we used the same cross-validation strategy, again in both groups, to generate predictions from “severity only” models (constructed using logistic regression), in which the only predictor available to the models was baseline symptom severity on the clinician-assessed Hamilton Rating Scale for Depression (HAMD; Hamilton, 1967; see Fig. S2 in the Supplemental Material), assessed using the 17-item GRID-HAMD (Williams et al., 2008).^4^ Outcome for all models was relapse, which was assessed retrospectively via the Structured Clinical Interview for DSM-IV (First et al., 1995) at five time points across the 24-month study period (1 month after acute intervention, and then 9, 12, 18, and 24 months after randomization; Kuyken et al., 2015a). See Figure 1 for a schematic summarizing the analytic pipeline.

#### Modeling via elastic-net regularized regression

Multivariable prognostic models were constructed using elastic-net regularized regression (ENRR; Zou & Hastie, 2005; see Fig. 1, Step 6). ENRR allows for the estimation of the predictive utility of a large number of variables, and its use has been demonstrated and extensively discussed in several previous predictive modeling efforts (Buckman et al., 2021; Chekroud et al., 2016, 2017; Cohen et al., 2020; Iniesta et al., 2016; Kim et al., 2019; Pearson et al., 2018; Webb et al., 2020). ENRR combines the L_1_ (LASSO penalty) and L_2_ (ridge regression penalty) penalization, which provides a hybrid of least absolute shrinkage and selection operator (LASSO) and ridge regression that thus addresses issues of correlated predictors and overfitting by shrinking coefficients of correlated predictors toward each other and by removing uninformative predictors from the model (Hastie et al., 2009). ENRR was implemented using the R package *glmnet* (Version 2.0-16; Friedman et al., 2010). Hyperparameter optimization (Fig. 1, Steps 3–5) was performed within each training sample using nested cross-validation. To reduce a source of potential bias (risk of overfitting because of information leakage from the test cases; Pearson et al., 2018) that can arise when a grid search is performed for hyperparameter setting in the context of cross-validation, we used three tuning loops (as suggested by a reviewer), 10-fold cross-validation (Friedman et al., 2010; Zou & Hastie, 2005, p. 310), and a small set of α values (.01, .5, .99) as implemented in the R package *beset* (Version 0.0.0.9409; Shumake, 2022) and described in Pearson et al. (2018) and McNamara et al. (2021). These three α values represent heavy weighting of the ridge penalty (α = .01), heavy weighting of the LASSO penalty (α = .99), or equal weighting (α = .5). The λ path of 100 possible values was generated using the *glmnet* package’s default calculation equation for λ path. In addition, the regularization parameter λ was selected using the one-standard-error rule, which helps to avoid overfitting and elevated Type I error (James et al., 2013; Waldmann et al., 2013). All analyses were performed in the R software environment (R Core Team, 2018); for additional information about packages used, see SM5 in the Supplemental Material.

##### Evaluating the models

Primary evaluation of model performance was performed via receiver-operating-characteristic (ROC) curves, which delineate the relative sensitivity (true-positive rate) and specificity (false-positive rate) of a model’s predictions at different thresholds. The area under the ROC curve (AUC) was used to quantify each model’s discrimination; AUCs of 0.5 indicate no or “chance” discrimination, and AUCs of 1 indicate perfect discrimination. In this context, because we are evaluating the outcome, “Did a relapse occur?” (yes/no), the AUC is equivalent to the concordance or c-statistic (Steyerberg et al., 2010). Another important aspect of model performance to evaluate is calibration (Van Calster et al., 2019); following recommendations based on sample size, we present only “weak calibration,” assessed via the calibration intercept and slope, with target values of 0 for the intercept (in which negative and positive values suggest overestimation and underestimation, respectively) and 1 for the slope (in which slopes > 1 indicate predictions that are too conservative and slopes < 1 indicate those that are too extreme).

We computed AUCs for the ENRR models’ factual predictions for patients in each treatment arm (ADM and MBCT; see Step 9^a^ of both Fig. 1 and Fig. S3 in the Supplemental Material). We also computed the AUC for each treatment arm for the depression-severity-at-baseline-only logistic regression models (HAMD) as a benchmark to compare against the more complex multivariable models. Within each treatment arm, we then compared these two AUCs using a one-tailed DeLong test for correlated ROC curves (DeLong et al., 1988) under the hypothesis that the multivariable models would outperform the benchmark models.

##### Evaluating prognostic utility

As noted in the results and described in detail in SM4 in the Supplemental Material, the internally cross-validated evaluation of the MBCT model’s factual predictions found that they were near chance and that they failed to noticeably outperform the HAMD model. We therefore focused our evaluation of prognostic utility on the ADM model alone under the rationale that in the absence of trustworthy information about MBCT prognosis, it would be rational to evaluate whether individuals who are predicted to have a high risk of relapse if they maintain ADM might have a better (relative) predicted outcome with a switch to MBCT. Likewise, we wanted to examine whether patients predicted to have good prognoses with ADM might be better advised to maintain the treatment regimen they are already following (i.e., ADM).

To evaluate the overall utility of the predictions generated by the ADM prognostic model in guiding treatment selection, we used two tertiles to divide the sample into three groups (Altman & Bland, 1994) on the basis of risk of relapse in ADM (good ADM prognosis, moderate ADM prognosis, and poor ADM prognosis). Sample sizes and descriptive statistics for the ADM prognoses (i.e., means, standard deviations, and ranges) for three groups, broken down by treatment received, are available in Table S3 in the Supplemental Material. Predictive utility of the ADM prognostic index was then evaluated by examining the time to relapse (in a survival analysis using Cox regression) and overall relapse rates over the 2-year follow-up. The independent variables were treatment condition (ADM, MBCT), ADM prognosis (both as a continuous variable and in categorical form: good, moderate, poor), and their interaction. For any significant interactions, the effects of treatment group were analyzed within each of the three prognostic categories.

## Results

### Model predicting relapse in the ADM treatment arm

**Fig. 2.**
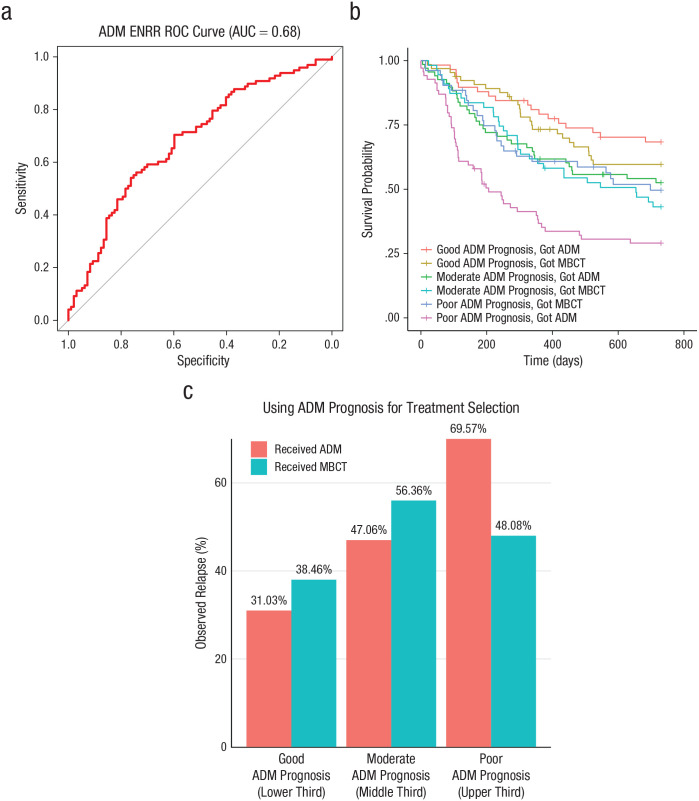
Probability of relapse in the ADM model. The graph in (a) shows the area under the receiver-operating-characteristic (ROC) curve (AUC), which delineates the relative sensitivity (true-positive rate) and specificity (false-positive rate) of the prognostic multivariable antidepressant medication (ADM) elastic-net model. The AUC (red line) is plotted against the straight gray line, which represents the threshold at which the model has no predictive utility. The gray line indicates the likelihood that someone above and below that threshold on the prognostic index has an equal likelihood of relapse. That is, the larger (farther away from the gray line) the AUC, the greater a model’s predictive utility. The graph in (b) plots the predicted survival curves for time (measured in days) to depressive relapse over the 2-year follow-up period for each ADM-prognosis group (poor, moderate, good) as a function of the treatment they received (mindfulness-based cognitive therapy [MBCT] or ADM). The graph in (c) shows the observed relapse rates over the 2-year follow-up as a function of the ADM relapse risk, separately by treatment received.

Using observed depressive relapse (yes/no) over 24 months to evaluate the factual predictions in the ADM model that had been made without the use of each patient’s own data, we found that the AUC for the ADM ENRR model was 0.68 (Fig. 2a), which was significantly better (one-tailed DeLong test: *z* = 2.80, *p* = .003) than that of the ADM HAMD comparison model (AUC = 0.54; see Fig. S4 in the Supplemental Material). The ADM ENRR model had a calibration intercept of −0.02 (in the direction of overestimation of relapse) and a calibration slope of 1.49 (which suggests overly conservative predictions at both ends of the risk spectrum). In contrast, the MBCT model (AUC = 0.54) did not outperform the HAMD comparison model (AUC = 0.52; *z* = 0.37, *p* = .35), a detailed description of which is available in Figure S5 in the Supplemental Material. Additional information regarding calibration for all models is available in SM4 in the Supplemental Material.

The specific variables that were retained and their associated coefficient weightings varied across the 10 ADM ENRR models that were generated. The key results of these models are summarized in Table 2. An expanded version of this table describing all 53 variables that were considered is provided in Table S4 in the Supplemental Material, and the analogous information for the 10 MBCT models is available in Table S5 in the Supplemental Material.

**Table 2.** Predictor Weightings for the ADM Prognostic Models Across 10-Fold Cross-Validation

Variable	No. of times selected	*M*	*SD*	Min	Max
MOPS level of parental abuse (low/high)^a^	10	0.34	0.16	0.03	0.57
Previous depressive episode chronicity^a,b^	10	−0.33	0.17	−0.60	−0.02
DPES Contentment	10	−0.08	0.06	−0.20	−0.01
DPES Joy	10	−0.05	0.038	−0.12	−0.003
DPES Love	10	−0.07	0.04	−0.14	−0.01
CERTS Negative Rumination	9	0.05	0.03	0	0.10
CERTS Unresolution	9	0.07	0.06	0	0.15
Previous suicide attempt^a^	8	0.10	0.09	0	0.26
Comorbidities	8	0.03	0.03	0	0.07
FFMQ Aware	8	−0.04	0.04	−0.11	0
CERQ Acceptance	8	0.04	0.05	0	0.14
GSE Self-Efficacy	7	−0.03	0.03	−0.08	0
Age of depression onset	6	−0.03	0.03	−0.08	0

Note: The table reports regression coefficients for the predictors in the ADM elastic-net prognostic models that were retained more than 50% of the time across the 10-fold cross-validation. In the model, all continuous variables entered were *z*-scored (*M* = 0, *SD* = 1), and dichotomous variables were set to −0.5 and +0.5. No. of times selected = number of times the variable was selected across the 10 cross-validations; min, max = minimum and maximum for variable’s coefficient value (includes zeros for when variable was not retained); ADM = antidepressant medication; MOPS = Measure of Parental Style (Parker et al., 1997); DPES = Dispositional Positive Emotion Scale (Shiota et al., 2006); CERTS = Cambridge-Exeter Repetitive Thought Scale (Barnard et al., 2007); FFMQ = Five Facet Mindfulness Questionnaire (Baer et al., 2006); CERQ = Cognitive Emotion Regulation Questionnaire (Garnefski et al., 2001); GSE = General Self-Efficacy Scale (Schwarzer & Jerusalem, 1995).

a Dichotomous variables (set to −0.5 and +0.5).

b Chronicity (no/yes) based on duration of previous depressive episode of 24 months or more.

Five baseline variables (from our set of 53) were retained as predictors of relapse across all 10 ADM ENRR models generated during the 10-fold CV procedure: level of child abuse, depression chronicity, and three subscales of the Dispositional Positive Emotions Scale (Shiota et al., 2006): Contentment, Joy, and Love. Higher levels of these positive emotions were associated with lower risk of relapse in ADM, whereas a history of child abuse was associated with increased risk of relapse. In the ADM models, having one’s most recent episode of depression be chronic (duration ≥ 24 months) was associated with reduced risk of relapse relative to people whose most recent episode was not. Two subscales of the Cambridge-Exeter Repetitive Thought Scale (Barnard et al., 2007) were retained in nine of the 10 models: Both Negative Rumination and Unresolution were associated with elevated risk of relapse in ADM. History of suicide attempt or attempts and number of comorbidities were both retained in eight of the 10 models and were associated with increased risk of relapse. Additional variables retained in more than 50% of the models are summarized in Table 2 and Table S4 in the Supplemental Material.

### Prognostic utility

We first verified that the outcome data for our analysis sample were comparable with that of the total PREVENT sample (Kuyken et al., 2015a). As in the full sample, survival times (*z* = −1.02; *p* = .31, HR [MBCT relative to ADM] = 0.86; 95% CI = [0.64, 1.15]) and relapse rates (MBCT = 47.1%, ADM = 50.3%) during the 24-month follow-up period in our analysis sample did not differ significantly between the two treatment conditions. In the survival analysis, in which we examined time to relapse with main effects for treatment and continuous ADM prognosis, there was a significant main effect of continuous ADM prognosis (*z* = 4.615; *p* < .001). We next compared observed outcomes across the two treatment conditions for individuals according to their ADM prognoses (i.e., good, moderate, poor; Fig. 2b).

The survival curves did not differ across treatments for individuals with good ADM prognoses (HR reflecting increased risk of relapse for those in MBCT vs. ADM = 1.34; 95% CI = [0.73, 2.45]; *p* = .35). The same was true for individuals with moderate ADM prognoses (HR = 1.19; 95% CI = [0.73, 1.96]; *p* = .48). In contrast, individuals with poor ADM prognoses had significantly reduced relapse risk (HR = 0.52; 95% CI = [0.32, 0.84]; *p* = .008) if they switched to MBCT instead of staying on ADM.

When comparing rates of participants who had actually relapsed by the end of the 2-year follow-up period, the same pattern emerged (Fig. 2c). There was a significant main effect of ADM prognosis on observed relapse rates, χ^2^(2) = 16.16, *p* < .001. As expected, the individuals with good ADM prognoses showed the lowest rates of relapse (35%), the group with moderate prognoses showed an intermediate relapse rate (51%), and the group with the poor prognoses showed the highest rate of relapse (60%). Relapse rates were low for individuals with good ADM prognoses regardless of which treatment they received (ADM = 31%, MBCT = 38%). Relapse rates did not differ significantly as a function of treatment assignment for this group, χ^2^(1) = 0.45, *p* = .50, or for those with moderate ADM prognoses (ADM = 47%, MBCT = 56%): χ^2^(1) = 0.71, *p* = .40. However, for individuals with poor ADM prognoses, relapse rates were significantly worse for participants who received ADM (70%) compared with participants who received MBCT (48%): χ^2^(1) = 4.86, *p* = .03. Finally, results from the sensitivity analyses that repeated the above analyses in a sample that included the 25 MBCT participants who had been excluded for not having attended at least four sessions of MBCT aligned with the results from the primary analysis sample (see SM2 in the Supplemental Material).

## Discussion

Clinical depression is a heterogeneous condition, which often runs a relapsing-and-remitting course across the life span and for which no single treatment has been shown to be effective for all patients (Fried, 2017; Fried & Nesse, 2015). A precision-medicine approach to depressive relapse prevention has potential utility in facilitating clinical choices between maintenance pharmacotherapy regimens and preventive psychosocial interventions such as MBCT.

We described a prognostic model that was developed using baseline data (demographic, clinical, and readily available psychological measures) from individuals who were randomly assigned to receive maintenance ADM following a successful course of acute treatment with ADM in an RCT comparing maintenance ADM with MBCT for relapse prevention. This ADM model (for a discussion of the predictors included in the model, see SM6 in the Supplemental Material), which predicts depressive relapse across a 24-month follow-up period, performed comparably with algorithms predicting acute remission response to antidepressants (Chekroud et al., 2016, 2017; Iniesta et al., 2016). We then generated ADM prognoses for the entire RCT sample (including participants randomly assigned to receive MBCT) to investigate whether the information from the ADM predictions might be helpful in deciding between staying on antidepressants or switching to preventive psychotherapy (MBCT). We observed a large difference in relapse rates for patients with poor ADM prognoses: 70% relapse in ADM versus 48% relapse in MBCT. In other words, patients with poor prognoses on ADM do not seem to simply be clinical nonresponders, but, rather, they may be individuals for whom MBCT represents a clinically beneficial alternative. Interpreted clinically, the findings suggest that if people present with a history of depression but do not report other risk factors, such as early abuse, anhedonia, rumination, and early onset, then ADM works well. However, our model suggests that when these other risk factors are present, it is worth considering MBCT because outcomes may be enhanced. This is consistent with other articles (Kuyken et al., 2016; Ma & Teasdale, 2004) that have suggested that for such individuals, there is more “grist for the MBCT mill” and possibly more motivation to engage in an active intervention such as MBCT (or indeed cognitive-behavioral therapy).

The survival model’s estimate of a 48% reduction in risk of relapse across the 24-month follow-up period (HR = 0.52) for patients with poor ADM prognoses who received MBCT versus ADM would suggest, if replicated, that such patients should pursue MBCT. The potential impact of the absolute observed difference in relapse rates (22%) for patients in the poor-ADM-prognosis subgroup who received ADM versus MBCT, however, is tempered by the fact that these individuals accounted for only one third of the sample. Yet the potential clinical utility of these findings may not necessarily be limited to this subgroup: Given the low relapse rates and lack of difference between treatments for patients with good ADM prognoses (31% ADM vs. 38% MBCT), such patients could be encouraged to select which relapse prevention strategy to pursue according to other factors. Clinically, our data indicate that treatment selection for depressive relapse prevention in individuals with recurrent depression who have a moderate to good ADM prognoses could be guided by factors such as patient preference, cost, and resource availability. Although resource availability may be a limiting factor, cost–benefit analyses have shown noninferiority of MBCT (Kuyken et al., 2015b), and some have even favored MBCT over ADM (Pahlevan et al., 2020). For individuals with poor prognoses on ADM, however, our data indicate that MBCT alongside tapering or cessation of medication to prevent relapse potentially confers a better clinical outcome and should be offered as an alternative to ADM. Recent systematic reviews and individual-participant meta-analyses suggest that combination relapse prevention, in which both medication continuation and preventive psychotherapy are provided, is superior to monotherapy and thus should also be considered for patients at higher risk for relapse.

Our study has a number of potential limitations. With the present data, we are unable to disaggregate the effects of MBCT from the tapering or discontinuation of ADM because they were both part of the MBCT protocol. We are also unable to comment on whether the effects are specific to MBCT or whether any effective alternative psychosocial intervention would offer potentially similar benefits for individuals with poor prognoses on maintenance ADM.

The utility of any model depends on its ability to generalize. The present algorithm was subjected to internal validation during variable selection and model building. The imputation of missing baseline data was not included in the cross-validation, but given the low number of missing data points, it is unlikely that this was a substantial source of bias. Previous work suggests that penalization and shrinkage methods may not provide as much protection as is assumed and that such methods (including ENRR) can produce unreliable clinical prediction models when sample sizes are small (Riley et al., 2021). Despite the internal cross-validation, we were not able to externally validate the model on a wholly independent sample because comparable sufficiently large trials evaluating the same preventive interventions with the same or a similar set of baseline measures are not currently available. This reflects the current state of precision medicine research (Cohen & DeRubeis, 2018), in which predictive models are too rarely subjected to proper external validation (Salazar de Pablo et al., 2021). Further external validation of the model^5^ and these results, when suitable data become available, will be an important next step before the translation of the current findings into firm treatment recommendations. Although we were fortunate to receive extensive reviewer feedback that allowed us to enhance our analytic approach, the many researcher degrees of freedom that remain represent potential threats to generalizability that merit caution and are worthy of further study.

Ideally, both the ADM and MBCT models would have been sufficiently robust to actively compare the two predictive indices to elucidate what works best for whom. However, our computed MBCT model did not perform above chance and was no better than a prediction model built solely on baseline depression severity scores. This lack of robust prediction within the MBCT model accords with the replicated finding that very few demographic, clinical, or psychological variables over and above baseline symptom severity appear to predict outcome to MBCT (Kuyken et al., 2016; Kuyken, Watkins, et al., 2010), which testifies to the intervention’s broad suitability. Second, in the present study, MBCT was combined with support for medication tapering or discontinuation, and it may be that the mixture of these two different intervention components (and possible associated effects of medication withdrawal) obscured any clear relations in the MBCT arm with the predictor variables included here.

The current findings represent a significant first step in the application of precision medicine to inform patient and clinician choice around optimal interventions for depressive relapse prevention. Additional work is needed to further validate the model reported here in wholly independent, yet-to-be-collected, large samples. The eventual success of this and similar personalized-medicine approaches to mental-health care will depend on the acquisition and dissemination of large-scale clinical data sets, which will allow for the development and validation of predictive models (Chekroud et al., 2017, 2021). The utility of these models must then be evaluated in prospective clinical trials (Delgadillo & Lutz, 2020), which have begun to emerge with promising results (e.g., Delgadillo et al., 2022; Lutz et al., 2022).

## Supplemental Material

sj-pdf-1-cpx-10.1177_21677026221076832 – Supplemental material for The Development and Internal Evaluation of a Predictive Model to Identify for Whom Mindfulness-Based Cognitive Therapy Offers Superior Relapse Prevention for Recurrent Depression Versus Maintenance Antidepressant Medication Click here for additional data file. Supplemental material, sj-pdf-1-cpx-10.1177_21677026221076832 for The Development and Internal Evaluation of a Predictive Model to Identify for Whom Mindfulness-Based Cognitive Therapy Offers Superior Relapse Prevention for Recurrent Depression Versus Maintenance Antidepressant Medication by Zachary D. Cohen, Robert J. DeRubeis, Rachel Hayes, Edward R. Watkins, Glyn Lewis, Richard Byng, Sarah Byford, Catherine Crane, Willem Kuyken, Tim Dalgleish and Susanne Schweizer in Clinical Psychological Science

## Transparency

*Action Editor:* Christopher G. Beevers

*Editor:* Kenneth J. Sher

*Author Contributions*

T. Dalgleish and S. Schweizer should be regarded as joint senior authors. Z. D. Cohen, T. Dalgleish, and S. Schweizer conceived of the secondary analyses. Z. D. Cohen analyzed the data. Z. D. Cohen, T. Dalgleish, and S. Schweizer wrote the manuscript. All coauthors commented on the final version of the manuscript. All of the coauthors approved the final manuscript for submission.

## Notes

1. We note that a recent systematic review of 56 studies and almost 40,000 subjects by De Zwart and colleagues (2019) reexamined the distinction between relapse and recurrence established by Frank et al. (1991) and concluded that “the idea that a recurrence of depressive symptoms shortly after their initial remission constitutes a ‘relapse’ of the previous episode, whereas their later recurrence is the first sign of an entirely new episode, is a model that lacks empirical support” (De Zwart et al., 2019, p. 544). Therefore, in this article, we use the term “relapse” to describe a return of depressive symptoms meeting criteria from the fourth edition of the *Diagnostic and Statistical Manual of Mental Disorders* (as assessed via Structured Clinical Interview for DSM-IV), regardless of when during the 24-month follow-up it occurred.

2. Although the terms “continuation” and “maintenance” are often used interchangeably in the literature, they are sometimes used more specifically (e.g., DeRubeis et al., 2019) to differentiate treatment following remission and up to recovery (continuation) from treatment past the point of recovery (maintenance). Here, we align with the trial’s main outcome article (Kuyken et al., 2015a) and use “maintenance” to describe all medication use during the study.

3. We note that in the PREVENT trial’s main outcome article (Kuyken et al., 2015a), “MBCT-TS” was used as the abbreviation for mindfulness-based cognitive therapy with support to taper or discontinue antidepressant treatment and that “m-ADM” was used as the abbreviation for maintenance antidepressant medication, but here these two conditions are simply abbreviated as MBCT and ADM, respectively.

4. From here forward, when we reference the “HAMD” (Hamilton Rating Scale for Depression) models (either for ADM or MBCT), we mean the simple “severity only” logistic regression comparison models that include only baseline HAMD, whereas when we reference the “ADM model” or the “MBCT model,” we mean the multivariable prognostic models constructed with elastic-net regularized regression.

5. See SM7 in the Supplemental Material for further details regarding a model constructed using the full ADM analysis sample that could be subjected to external validation in future efforts.
