# Supplementary material for: The Development and Internal Evaluation of a Predictive Model to Identify for Whom Mindfulness-Based Cognitive Therapy Offers Superior Relapse Prevention for Recurrent Depression Versus Maintenance Antidepressant Medication
Source: Clin Psychol Sci. 2022 Apr 29;11(1):59–76. doi: 10.1177/21677026221076832 (PMC7614103; doi:10.1177/21677026221076832)
Supplement: sj-pdf-1-cpx-10.1177_21677026221076832 – Supplemental material for The Development and Internal Evaluation of a Predictive Model to Identify for Whom Mindfulness-Based Cognitive Therapy Offers Superior Relapse Prevention for Recurrent Depression Versus Maintenance Antidepressant Medication [file sj-pdf-1-cpx-10.1177_21677026221076832.pdf]

Supplemental Material

The development and internal evaluation of a predictive model to identify for whom Mindfulness-Based Cognitive Therapy (MBCT) offers superior relapse prevention for recurrent depression versus maintenance antidepressant medication

Zachary D. Cohen, PhD

Robert J. DeRubeis, Professor

Rachel Hayes, PhD

Edward. R. Watkins, Professor

Glyn Lewis, Professor

Richard Byng, Professor

Sarah Byford, Professor

Catherine Crane, PhD

Willem Kuyken, Professor

Tim Dalgleish*, Professor

Susanne Schweizer*, PhD

*Joint senior authors

Table of Contents

Table of Contents *2*

SM1 – Participant Exclusion *3*

Figure S1 .... *3*

SM2 - Sensitivity Analyses *4*

SM3 - Descriptive data for predictor variables at baseline *7*

ADM vs MBCT Sample 7

Excluded vs Included (Analysis) Sample 15

SM4 - Model Construction *23*

Cross-validation 23

Modeling ADM Prognosis 25

Modeling MBCT Prognosis 26

Variable Selection Results 30

SM5 - R Packages *40*

SM6 - Discussion of Model Components *42*

SM7 – Table S7. ADM Model from Full ADM Analysis Sample *46*

Supplementary references 48

SM1 – Participant Exclusion

Figure S1

Pipeline for Participant Exclusion ~ Data Exclusion Pipeline

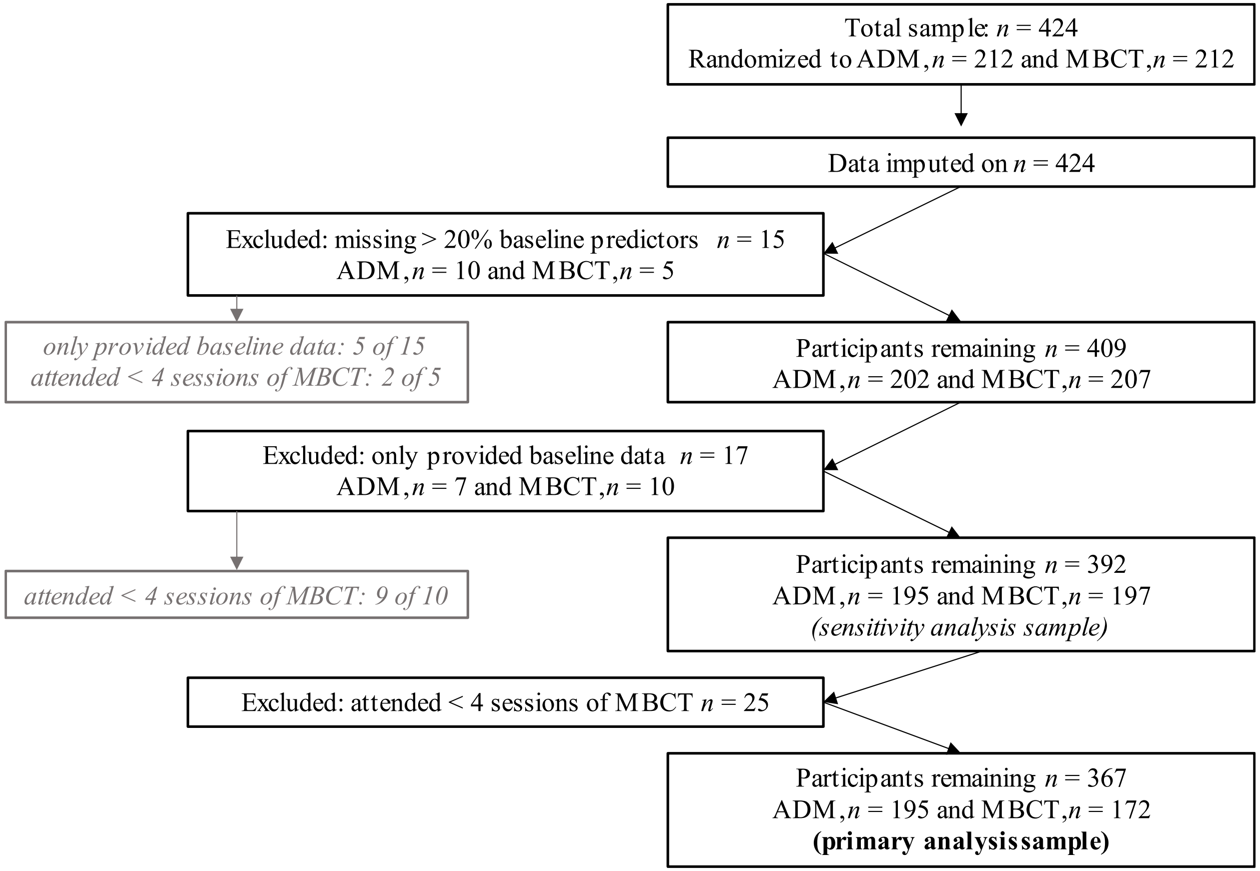

Note. MBCT = Mindfulness-Based Cognitive Therapy; ADM = Antidepressant medication

SM2 - Sensitivity Analyses

Because we did not end up using the MBCT prediction model in our treatment selection analyses, all analyses were repeated including the 25 MBCT participants that were initially excluded from the main analyses based on lack of treatment dose (completing fewer than four MBCT sessions). Consistent with the main analyses, the ADM AUC continued to offer significantly greater predictive utility relative to the HAMD-only model AUC, $z = 2.80$, $p = .003$. This was still not the case for the MBCT model, where there remained no significant difference in AUCs relative to the HAMD-only model, $z = 0.34$, $p = .37$ (Figure S2).

Figure S2

Receiver Operating Characteristic (ROC) Curves for Probability of Relapse within the ADM and MBCT Groups for the Sensitivity Analyses

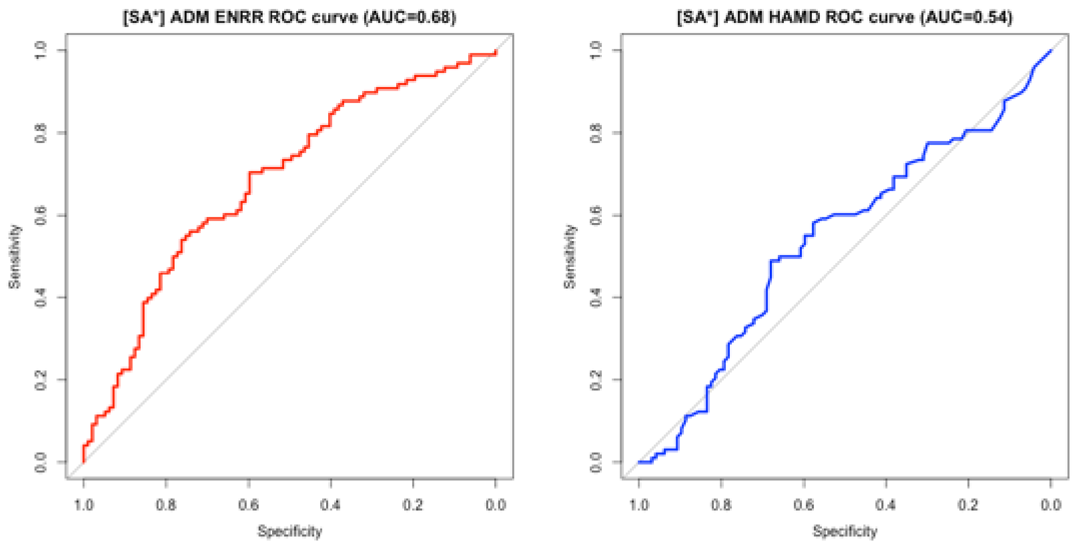

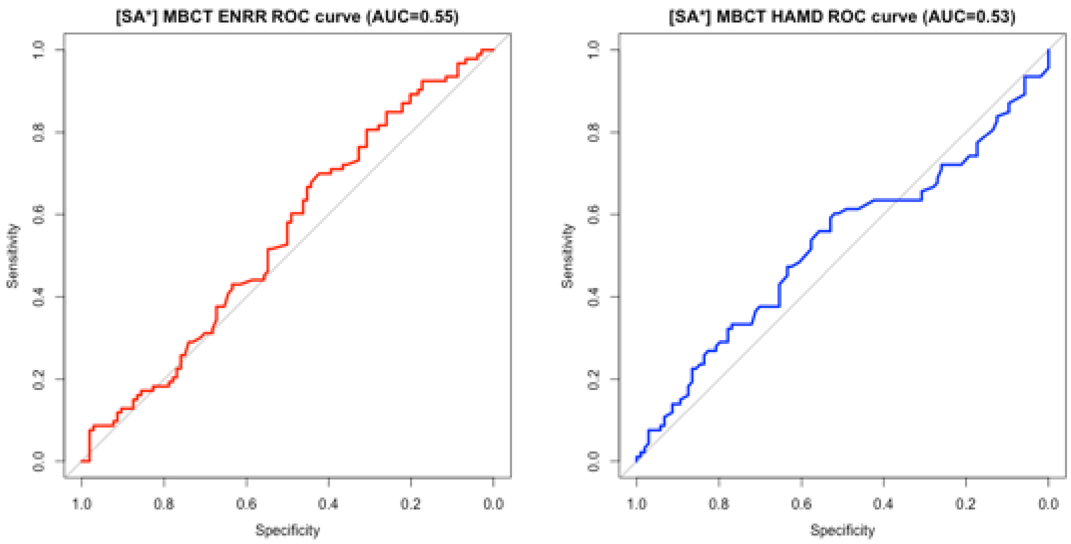

Note. The panels show the Area Under receiver operating characteristic (ROC) Curves (AUC) for the prediction models. The curves delineate the relative sensitivity (true positive rate) and specificity (false positive rate) of the prediction models. The left panels (in red) show the AUCs for multi-variable elastic net (ENRR) models predicting the rate of relapse over 24 months in ADM (top) and MBCT (bottom). The right panels (in blue) shows the AUCs for the comparison models using baseline Hamilton Rating Scale for Depression (HAMD) as the only predictor across the two treatment arms, again for ADM (top) and MBCT (bottom). The AUCs are plotted against the straight grey line, which represents the threshold at which the model has no predictive utility. The grey line delineates the likelihood that someone above and below that threshold on the prognostic index has an equal likelihood of relapse. That is, the larger (further away from the grey line) the AUC the greater a model's predictive utility. SA* = Sensitivity Analysis.

In the survival analysis examining time-to-relapse with main effects for treatment and continuous ADM prognosis for the sensitivity sample, as in the main analysis sample, there was a significant main effect of continuous ADM prognosis ($z = 4.237$; $p < .001$). We next compared observed outcomes across the two treatment conditions for individuals

according to their ADM-prognosis. As in the main analysis sample, the predicted survival curves did not differ across treatments for those with good ADM prognoses (hazard ratio reflecting increased risk of relapse for those in MBCT vs. ADM = 1.27; 95%CI, 0.70 to 2.31; p = .43), or for those with moderate ADM prognoses (hazard ratio = 1.11; 95%CI, 0.69 to 1.79; p = .66). In contrast to those with good and poor prognoses, again aligning with the main analysis sample results, those with poor ADM prognoses had significantly reduced relapse risk (hazard ratio = 0.59; 95%CI, 0.38 to 0.93; p = .023) if they switched to MBCT instead of staying on ADM.

As with the survival sensitivity analyses, the results of the sensitivity analyses of numbers relapsed by the end of follow up rates were aligned with the main analyses. There was the expected significant main effect of prognostic sub-group on numbers relapsed, X^2 (2) = 17.98, $P < .001$. Investigating numbers relapsed across each prognostic sub-group, again revealed no significant effects in the sub-groups with moderate, X^2 (1) = 0.41, $P = .52$ and good X^2 (1) = 0.28, $P = .60$, ADM-prognoses. The 18% difference in numbers relapsed by the end of follow-up (51% MBCT vs. 69% ADM) in the poor ADM-prognosis group for the sensitivity analysis sample was smaller than the 22% difference observed in the main analysis sample, and was no longer significant (X^2 (1) = 3.60, $P =$ .052).[*]

[*] As humorously but depressingly illustrated by Matthew Hankin's famous blog post (link below) summarizing more than 500 unique phrases that have been used in peer-reviewed journal articles to inaccurately describe non-significant results, incorrectly describing "marginally significant" (i.e., non-significant) results is a problem in the scientific literature (Olsson-Collentine et al., 2019). In the effort to contribute science's self-correction, we must thank Reviewer #2 for correcting our embarrassing misstep of describing one of our results as "trending towards significance" in our initial submission, and error which we have corrected and will never again commit.

https://mchankins.wordpress.com/2013/04/21/still-not-significant-2/

SM3 - Descriptive data for predictor variables at baseline

ADM vs MBCT Sample

Descriptive data for the predictor variables, prior to imputation, at baseline in the analysis sample (*N*=367) broken down by treatment group (ADM vs MBCT) is provided in Table S1, along with group comparisons. There was a significantly greater proportion of women in the ADM group (82% vs 69%), and ADM participants, on average, were 2.5 years younger, reported 0.2 more comorbid diagnoses, and had a lower probability that their most recent episode of depression was chronic (>24 months in duration), at baseline, compared to the MBCT group (19% vs 31%, respectively; see Table S1 for more details).

Table S1

Predictor Variables at Baseline in the Primary Analysis Sample, Broken Down by Treatment Group

Predictor	ADM (*N* = 195)	MBCT (*N* = 172)	Continuous: Mean difference (t-stat) Categorical: χ^2	*P* Value
**Demographic characteristics**				
Age (years)			**1.99**	**.048**
Mean (*sd*)	48.77 (12.69)	51.30 (11.56)		
Range	20-79	24-78		
**Female (%)**	160 (82)	118 (69)	**8.28**	**.004**
Education[†]			–1.47[††]	.14[††]
No educational qualification	10 (5)	10 (6)		
O levels or GCSEs	38 (20)	24 (14)		
AS and A levels (UK Advanced Level)	26 (13)	15 (9)		
Vocational training/qualification	64 (33)	56 (33)		
University Bachelor's degree	32 (17)	44 (26)		
University Master's degree	9 (5)	9 (5)		
University professional training/PhD	14 (7)	12 (7)		
Relationship			0.33	.57
No (Single/Divorced/Widowed)	67 (34)	65 (38)		
Yes (Married/Civil partnership/Cohabiting)	128 (66)	107 (62)		
Employed* (unemployed vs. full- or part-time)	119 (61)	98 (57)	0.46	.50
**Clinical characteristics**				
Clinician-rated depressive symptoms (HAMD)	4.62 (4.31)	4.76 (4.27)	0.31	.75
Mean (*sd*)	0-20	0-19		
Range				

Self-reported depressive symptoms (BDI-II)			–0.76	.45
Mean (*sd*)	14.39 (10.08)	13.59 (10.24)		
Range	0-42	0-48		
Age of onset			–0.20	.84
Mean (*sd*)	25.16 (12.30)	24.91 (11.82)		
Range	6-65	5-67		
**Chronicity (previous depressive episode $\geq$ 24months)**	38 (19)	53 (31)	**5.69**	**.02**
Previous psychological treatment	98 (52)	84 (49)	0.17	.68
Previous suicide attempt	49 (25)	33 (19)	1.53	.22
Family history of depression	90 (50)	85 (53)	0.13	.72
**Number of comorbid diagnoses**			**–2.59**	**.01**
Mean (*sd*)	0.68 (0.94)	0.44 (0.77)		
Range	0-5	0-3		
**Psychological mechanisms**				
Five-Facets Mindfulness Questionnaire (FFMQ)				
Observe			0.19	.85
M (*sd*)	24.11 (5.63)	24.23 (5.69)		
Range	11-37	8-39		
Describe			0.38	.71
M (*sd*)	26.08 (7.14)	26.35 (6.62)		
Range	8-40	10-40		
Aware			0.34	.73
M (*sd*)	24.01 (5.28)	24.20 (5.62)		
Range	10-40	10-39		
Non-Judging			0.16	.87
M (*sd*)	24.87 (6.35)	24.98 (6.93)		
Range	10-40	8-39		
Non-Reactivity			1.58	.11
M (*sd*)	19.29 (4.59)	20.10 (5.24)		

Range	10-31	7-35		
Self-Compassion Scale (SCS)				
Self-Kindness			0.01	.99
M (*sd*)	12.57 (3.96)	12.58 (4.35)		
Range	5-25	5-25		
Self-Judgement			0.39	.69
M (*sd*)	11.77 (3.91)	11.93 (4.02)		
Range	5-25	5-24		
Common Humanity			–0.50	.62
M (*sd*)	11.79 (3.81)	11.58 (3.85)		
Range	4-20	4-20		
Isolation			–0.49	.63
M (*sd*)	9.58 (3.32)	9.40 (3.48)		
Range	4-20	4-20		
Mindfulness			–0.03	.98
M (*sd*)	11.79 (3.14)	11.78 (3.39)		
Range	4-20	4-20		
Over-Identification			0.42	.67
M (*sd*)	9.22 (3.11)	9.37 (3.43)		
Range	4-19	4-20		
Compassion for others			0.65	.51
M (*sd*)	27.15 (3.32)	27.38 (3.36)		
Range	16-33	11-33		
Dispositional Positive Emotions Scale (DPES)				
Joy			–1.07	.29
M (*sd*)	17.23 (4.34)	16.74 (4.38)		
Range	6-30	6-28		
Contentment			–0.29	.77
M (*sd*)	14.18 (3.93)	14.05 (4.15)		
Range	5-25	5-25		

Love			–0.62	.53
M (*sd*)	19.22 (4.66)	18.92 (4.23)		
Range	6-30	9-29		
Compassion			–0.63	.53
M (*sd*)	21.36 (3.00)	21.16 (3.22)		
Range	12-25	13-25		
Awe			1.13	.26
M (*sd*)	19.25 (4.45)	19.786 (4.09)		
Range	8-30	9-30		
Curiosity			1.10	.27
M (*sd*)	14.39 (3.20)	14.76 (3.19)		
Range	4-20	5-20		
Cognitive Emotion Regulation Questionnaire (CERQ)				
Catastrophizing			1.35	.18
M (*sd*)	8.54 (2.95)	8.99 (3.39)		
Range	4-18	4-18		
Rumination			0.44	.66
M (*sd*)	12.04 (3.55)	12.20 (3.42)		
Range	4-20	5-20		
**Other-blame**			1.20	.23
M (*sd*)	7.41 (2.46)	7.75 (3.00)		
Range	4-20	4-18		
Self-blame			0.91	.36
M (*sd*)	10.72 (3.39)	11.05 (3.64)		
Range	4-20	4-20		
Acceptance			–0.08	.94
M (*sd*)	11.76 (3.02)	11.74 (3.03)		
Range	6-20	5-19		
Positive Refocusing			–1.52	.13
M (*sd*)	8.10 (3.16)	7.59 (3.26)		

Range	4-18	4-18		
Positive Reappraisal			0.54	.59
M (*sd*)	10.05 (3.78)	10.28 (4.25)		
Range	4-20	4-20		
Putting into Perspective			–0.31	.76
M (*sd*)	10.95 (3.41)	10.83 (3.89)		
Range	4-20	4-20		
Refocus on Planning			0.63	.53
M (*sd*)	10.64 (3.32)	10.87 (3.71)		
Range	4-20	4-20		
Cambridge-Exeter Repetitive Thought Scale (CERTS)				
Negative Rumination			0.56	.58
M (*sd*)	73.18 (15.37)	74.08 (15.10)		
Range	23-100	20-100		
Positive Rumination			1.40	.16
M (*sd*)	22.43 (5.84)	23.28 (5.71)		
Range	8-40	8-38		
Constructive Rumination			1.38	.17
M (*sd*)	10.91 (3.04)	11.35 (3.09)		
Range	4-20	4-20		
Unresolution			0.01	.99
M (*sd*)	12.31 (2.70)	12.31 (2.97)		
Range	4-19	4-20		
Moving On			–0.79	.43
M (*sd*)	7.83 (1.76)	7.68 (1.74)		
Range	3-13	4-12		
**Other**				
Level of parental abuse (MOPS)			0.47	.49
Low	93 (48)	90 (52)		
High	100 (52)	82 (48)		

Self-Efficacy (GSE)			–0.05	.96
Mean (*sd*)	32.31 (7.77)	32.27 (8.18)		
Range	13-50	10-50		
Stigmatisation (SN)			–0.39	.70
Mean (*sd*)	20.88 (6.38)	20.60 (7.15)		
Range	7-35	7-35		
Recognizing warning signs (WS)			1.25	.21
Mean (*sd*)	18.26 (5.83)	19.01 (5.55)		
Range	6-30	6-30		
Relationship Satisfaction (RS)			0.88	.38
Mean (*sd*)	26.50 (6.71)	27.11 (6.28)		
Range	7-35	9-35		
Preference for MBCT			–0.46	.64
	4.51 (0.67)	4.48 (0.72)		
	2-5	1-5		
Preference for ADM			0.50	.61
	3.10 (1.10)	3.16 (1.09)		
	1-5	1-5		
Preference for Therapy Type			–0.83	.41
	1.80 (1.07)	1.70 (1.07)		
	1-5	1-5		

Note. HAMD = Hamilton Depression Rating Scale (Hamilton, 1967), assessed using the 17-item GRID-HAMD (Williams et al., 2008); BDI-II = Beck Depression Inventory Version-II (Beck et al., 1996); MOPS = Measure of Parenting Style (Parker et al., 1997); GSE = General Self-Efficacy Scale (Schwarzer et al., 1995); SN = Stigmatization and Normalization (bespoke questions); WS = Warning signs (bespoke questions); RS= Relationship satisfaction (bespoke questions). † Education, which was assessed and imputed as an ordered categorical variable, was transformed into a continuous variable following imputation as follows: 0 = No educational qualification, 1 = O levels or GCSEs, 2 = AS and A

levels, 3 = Vocational training, 4 = University Bachelor's degree, 5 = University Master degree, 6 = University professional training. †† The reported group difference test comparing excluded vs. included for Education is for the numeric version of the variable via a mean difference test (Two Sample Student's t-Test). * Employed was defined as a categorical variable where Yes (employed=1) was defined as full- or part-time employed, and No (unemployed=0) also included the following categories: retired, voluntary working, housewife, househusband, homemaker, full time mum/dad, student, “not applicable”, at home, and carer for family member. Individuals were asked to complete all measures (except for the MOPS) with respect to the previous two weeks. All scales except for the HAMD, BDI-II, and MOPS were scored on a 5-point Likert scale irrespective of their original scoring range. The scaling was standardized to facilitate interpretation from factor analyses and similar computations planned for the trial. The labels of the original scales were maintained. Further details on the psychological predictor variables are presented in Table 1.

Excluded vs Included (Analysis) Sample

Descriptive data for the predictor variables, prior to imputation, at baseline for the excluded and included (i.e., main analysis) samples, are provided in Table S2, along with group comparisons and information on missingness. Relative to the analysis sample, excluded participants were, on average, four years younger, had 0.3 more comorbid diagnoses, and reported lower scores on the Dispositional Positive Emotions Scale Curiosity subscale, Self-Compassion Scale Isolation subscale, and the Five-Facets Mindfulness Questionnaire Describe subscale.

Table S2

Predictor Variables at Baseline

Predictor	Excluded (*n* = 57)	Included (*n* = 367)	Continuous: Mean difference (t-stat) Categorical: χ^2	*P* Value
Demographic				
**Age (years)**			**2.23**	**.03**
Mean (*sd*)	46.07 (12.42)	49.96 (12.22)		
Range	22-73	20-79		
Female	47 (82)	278 (76)	0.89	.34
Education[†]	[a]	[b]	0.68[††]	.50[††]
No educational qualification	0 (0)	20 (6)		
O levels or GCSEs	19 (37)	62 (17)		
AS and A levels (UK Advanced Level)	1 (2)	41 (11)		
Vocational training/qualification	14 (27)	120 (33)		
University Bachelor's degree	13 (25)	76 (21)		
University Master's degree	2 (4)	18 (5)		
University professional training/Phd	3 (6)	26 (7)		
Relationship	[c]		1.46	.23
No (Single/Divorced/Widowed)	25 (45)	132 (36)		
Yes (Married/Civil Partnership/Cohabiting)	30 (55)	235 (64)		
Employed* (unemployed vs. full- or part-time)	24 (44)[d]	217 (59)	3.57	.06
**Clinical characteristics**				
Clinician-rated depressive symptoms (HAMD)			–0.15	.88
Mean (*sd*)	4.77 (4.61)	4.68 (4.29)		
Range	0-19	0-20		
Self-reported depressive symptoms (BDI-II)			–0.49	.62
Mean (*sd*)	14.78 (10.05)[e]	14.01 (10.15)		
Range	0-37	0-48		

Age of depression onset			1.49	.14
Mean (*sd*)	22.53 (10.41)	25.04 (12.06)[f]		
Range	4-50	5-67		
Chronicity (previous depressive episode ≥24 months)	11 (19)	91 (25)	0.54	.46
Previous psychological treatment	29 (55)[g]	182 (50)	0.21	.65
Previous suicide attempt	19 (35)[e]	82 (22)	3.58	.06
Family history of depression	24 (51) [h]	175 (51)[i]	9.68e-30	1.00
**Number of comorbid diagnoses**			**–2.28**	**.02**
Mean (*sd*)	0.86 (1.06)	0.57 (0.87)		
Range	0-4	0-5		
**Psychological Mechanisms**				
**Five-Facets Mindfulness Questionnaire (FFMQ)**	[j]	[k]		
Observe			1.07	.29
M (sd)	23.20 (5.60)	24.17 (5.65)		
Range	11-39	8-39		
**Describe**			**2.10**	**.04**
M (sd)	23.93 (5.67)	26.20 (6.90)		
Range	10-36	8-40		
Aware			0.90	.37
M (sd)	23.32 (5.24)	24.10 (5.44)		
Range	15-37	10-37		
Non-Judging			–0.49	.62
M (sd)	25.45 (6.79)	24.92 (6.62)		
Range	12-39	8-40		
Non-Reactivity			0.82	.42
M (sd)	19.05 (4.02)	19.67 (4.92)		
Range	11-29	7-35		
**Self-Compassion Scale (SCS)**	[g]	[b]		
Self-Kindness			0.50	.62
M (sd)	12.24 (3.89)	12.58 (4.14)		
Range	5-20	5-25		

Self-Judgement			0.68	.50
M (sd)	11.40 (4.03)	11.84 (3.96)		
Range	5-25	5-25		
Common Humanity			0.66	.51
M (sd)	11.29 (3.44)	11.69 (3.83)		
Range	5-19	4-20		
**Isolation**			**2.23**	**.03**
M (sd)	8.29 (2.72)	9.50 (3.39)		
Range	4-15	4-20		
Mindfulness			0.31	.76
M (sd)	11.62 (3.37)	11.78 (3.25)		
Range	4-18	4-20		
Over-Identification			–0.03	.97
M (sd)	9.31 (2.86)	9.29 (3.26)		
Range	4-20	4-20		
Compassion for others			0.21	.83
M (sd)	27.14 (3.26)	27.26 (3.34)		
Range	19-34 [g]	11-33 [k]		
**Dispositional Positive Emotions Scale (DPES)**				
Joy			–0.37	.71
M (sd)	17.26 (3.64)	17.00 (4.36)		
Range	9-24	6-30		
Contentment			0.07	.94
M (sd)	14.07 (3.64)	14.12 (4.03)		
Range	7-23	5-25		
Love			0.84	.40
M (sd)	18.48 (3.77)	19.08 (4.46)		
Range	9-27	6-30		
Compassion			–0.27	.79
M (sd)	21.40 (3.86)	21.27 (3.10)		
Range	5-25	12-25		

Awe			1.76	.08
M (sd)	18.26 (4.18)	19.50 (4.29)		
Range	6-28	8-30		
**Curiosity**			**2.12**	**.04**
M (sd)	13.48 (2.83)	14.57 (3.19)		
Range	6-20	4-20		
**Cognitive Emotion Regulation Questionnaire (CERQ)**	[g]	[f]		
Catastrophizing			–0.72	.47
M (sd)	9.12 (2.77)	8.75 (3.17)		
Range	5-15	4-18		
Rumination			1.00	.32
M (sd)	11.55 (3.47)	12.12 (3.49)		
Range	6-20	4-20		
Other-blame			0.37	.71
M (sd)	7.40 (2.60)	7.57 (2.73)		
Range	4-14	4-15		
Self-blame			–0.70	.49
M (sd)	11.29 (4.45)	10.87 (3.51)		
Range	4-20	4-20		
Acceptance			–0.72	.47
M (sd)	12.12 (3.91)	11.75 (3.02)		
Range	6-20	5-20		
Positive Refocusing			0.29	.77
M (sd)	7.71 (2.26)	7.86 (3.22)		
Range	4-14	4-15		
Positive Reappraisal			0.54	.59
M (sd)	9.81 (3.55)	10.16 (4.01)		
Range	4-19	4-20		
Putting into Perspective			–0.23	.82
M (sd)	11.02 (2.62)	10.89 (3.64)		
Range	5-18	4-20		

Refocus on Planning			0.86	.39
M (sd)	10.26 (3.16)	10.75 (3.51)		
Range	5-18	4-20		
**Cambridge-Exeter Repetitive Thought Scale (CERTS)**	l			
Negative Rumination		m	0.98	.33
M (sd)	71.23 (13.15)	73.61 (15.23)		
Range	29-91	20-100		
Positive Rumination		m	0.32	.75
M (sd)	22.53 (6.10)	22.83 (5.79)		
Range	8-34	8-40		
Constructive Rumination		n	0.86	.39
M (sd)	10.70 (2.86)	11.12 (3.07)		
Range	5-19	4-20		
Unresolution		k	–1.17	.24
M (sd)	12.84 (2.37)	12.31 (2.83)		
Range	6-17	4-20		
Moving on		n	–1.93	.055
M (sd)	8.30 (1.66)	7.76 (1.75)		
Range	5-13	3-13		
**Other**				
Level of parental abuse (MOPS)	e	k	0.25	.62
Low	27 (55)	183 (50)		
High	22 (45)	182 (50)		
Self-Efficacy (GSE)			1.24	.22
Mean (*sd*)	30.76 (6.95)[o]	32.29 (7.96)[k]		
Range	16-50	10-50		
Perceived Stigmatisation (SN)			–0.99	.32
Mean (*sd*)	21.83 (6.67)[g]	20.75 (6.74)[c]		
Range	7-34	7-35		
Recognizing Warning Signs (WS)			–0.56	.58
Mean (*sd*)	19.12 (4.24)[g]	18.61 (5.70)[f]		

Range	12-28	6-30		
Relationship Satisfaction (RS)			–0.95	.34
Mean (*sd*)	27.79 (6.29)[c]	26.78 (6.51)		
Range	12-35	7-35		
Preference for MBCT			1.08	.28
Mean (*sd*)	4.38 (0.87)[c]	4.49 (0.69)[n]		
Range	2-5	1-5		
Preference for ADM			0.11	.91
Mean (*sd*)	3.11 (1.10)[c]	3.13 (1.09)[n]		
Range	1-5	1-5		
Preference for Therapy Type			–1.83	.07
Mean (*sd*)	2.04 (1.10)[c]	1.75 (1.07)[n]		
Range	1-5	1-5		

Note. Data are n (%) unless otherwise specified. Missing cases resulted in the following *n* for the following variables. [a] *n* = 52; [b] *n* = 363; [c] *n* = 55; [d] *n* = 54; [e] *n* = 49; [f] *n* = 366; [g] *n* =42; [h] *n* =47; [i] *n* = 340; [j] *n* = 44; [k] *n* = 365; [l] *n* = 43; [m] *n* = 362; [n] *n* = 364; [o] *n* = 45. HAMD = Hamilton Depression Rating Scale (Hamilton, 1967), assessed using the 17-item GRID-HAMD (Williams et al., 2008); BDI-II = Beck Depression Inventory Version II (Beck et al., 1996); MOPS = Measure of Parenting Style (Parker et al., 1997); GSE = General Self-efficacy Scale (Schwarzer et al., 1995); SN = Stigmatization and Normalization (bespoke questions); WS = Warning signs (bespoke questions); RS= Relationship satisfaction (bespoke questions). [†] Education, which was assessed and imputed as an ordered categorical variable, was transformed into a continuous variable following imputation as follows: 0 = No educational qualification, 1 = O levels or GCSEs, 2 = AS and A levels, 3 = Vocational training, 4 = University Bachelor's degree, 5 = University Master degree, 6 = University professional training. [††] The reported group difference test comparing excluded vs. included for Education is for the numeric version of the variable via a mean difference test (Two Sample

Student's t-Test). * Employed was defined as a categorical variable where Yes (employed) was defined as full- or part-time employed, and No (unemployed) also included the following categories: retired, voluntary working, housewife, househusband, homemaker, full time mum/dad, student, "not applicable", at home, and carer for family member. Preference questions were: "How do you feel about the possibility of being in an MBCT group" and "How do you feel about remaining on your ADMs", both scored on a 5-point Likert scale with anchors of 1 = not positive at all and 5 = extremely positive, and "Do you have a preference for a group", also scored on a 5-point Likert scale with anchors of 1 = MBCT, 3 = no pref, and 5 = continue on ADM.

SM4 - Model Construction

Cross-validation

Most treatment selection work in mental health has suffered from two potential limitations related to the sample sizes usually available in randomized controlled trials (Lorenzo-Luaces et al., 2020) and a lack of separate test/validation samples (Cohen et al., 2021). First, questions regarding a model's generalizability beyond the sample in which it was built (or population from which the sample was drawn) are not easily addressed without a held-out sample in which the model can be evaluated. In an ideal world, every predictive model would be evaluated in a completely separate test sample. Here, this was not possible, as no other study comparing ADM to MBCT has measured a comparably inclusive set of potential predictors as used in our analyses. Thus, although the variable selection approach and weight setting approach we employed was designed to improve generalizability and reliability (c.f., Riley et al., 2021), the extent to which our final model would generalize to a new population is unknown.

The second issue with most analytic efforts that rely on small RCT samples (in which held-out test samples are not practical) is the risk of overconfidence due to "double-dipping", which arises when a model is evaluated within the same sample in which it was constructed (Fiedler, 2011; Hastie et al., 2009). When a truly separate sample is unavailable, one approach to avoid double-dipping is to perform split-halves analysis, in which the sample is split into two halves, one of which is used to create the model, while the other is completely held out to evaluate the model. Given our small sample, a split-halves approach was not feasible. To maximize the sample size available for model creation while simultaneously ensuring that data from the individual for whom predictions were being generated did not contribute to the predictive model, we therefore

employed K-fold cross-validation (specifically, 10-fold), as described in the main manuscript.

Figure S3

Schematic of Cross-validation Procedure for Producing MBCT Predictions for the Full Analysis Sample

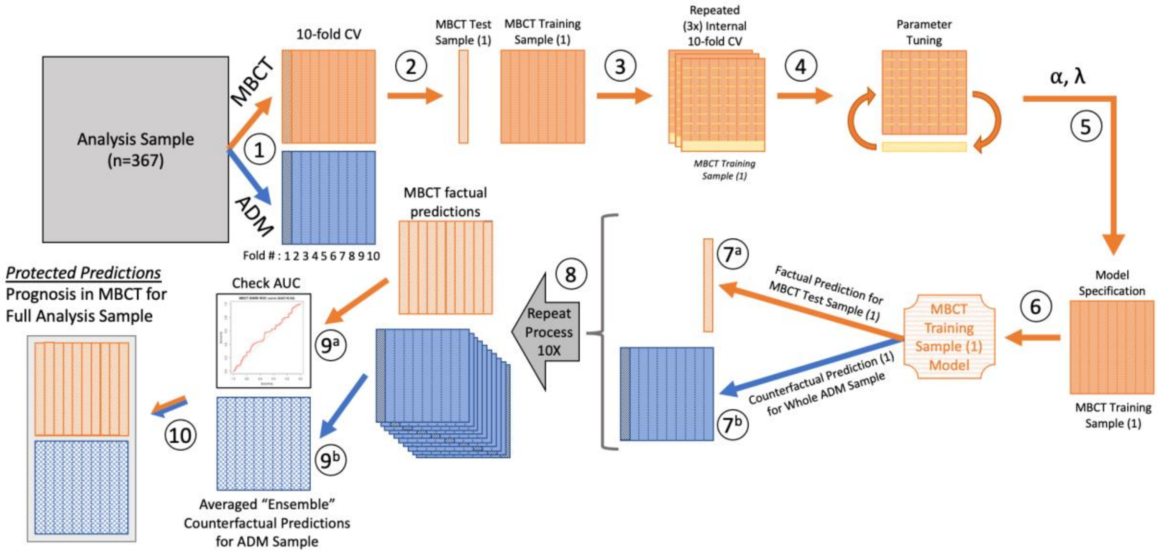

Figure S3: Step 1 (10-fold cross-validation [CV]): The main analysis sample was separated into MBCT and ADM samples, which were then split into ten sub-groups, balanced on outcomes. Step 2: The MBCT sample was separated into its first train-test samples, with the first of the ten sub-groups held out as MBCT Test Sample (1), and the other nine sub-groups comprising MBCT Training Sample (1). Steps 3 and 4: MBCT Training Sample (1) was then itself split into ten sub-groups, and parameter tuning was performed using internal 10-fold cross-validation; this entire process was repeated 3 times using different random permutations of the internal 10-fold CV of MBCT Training Sample (1). Step 5 (hyperparameter optimization): The optimal alpha (α) and lambda (λ) were selected and used in Step 6 (Model Specification), in which Elastic Net Regularized Regression (ENRR) was applied to the entire MBCT Training Sample (1) to derive MBCT Training Sample (1) Model. Step 7[a]: This model was then used to generate factual predictions for held-out MBCT Test Sample (1), and to generate counterfactual predictions (Step 7[b]) for the entire ADM Sample. Step 8: Steps 2-7 were then repeated nine more times to complete the 10-fold CV. Step 9[a]: The resulting set of (protected) factual predictions for the entire MBCT sample (likelihood of relapse in MBCT) were then evaluated using the Area Under the Receiver Operating Characteristic Curve (AUC). Step 9[b]: The set of ten (protected) counterfactual predictions for each individual in the ADM sample (likelihood of relapse if they had received MBCT) were averaged, resulting in a set of Averaged “Ensemble” Counterfactual Predictions for the ADM sample. Step 10: The MBCT and ADM samples and their MBCT predictions were then re-combined, resulting in protected Prognoses in MBCT for the Full Analysis Sample.

Modeling ADM Prognosis

Figure S4

Area Under the Receiver Operating Characteristic (ROC) Curve (AUC) for ADM Prognostic Models

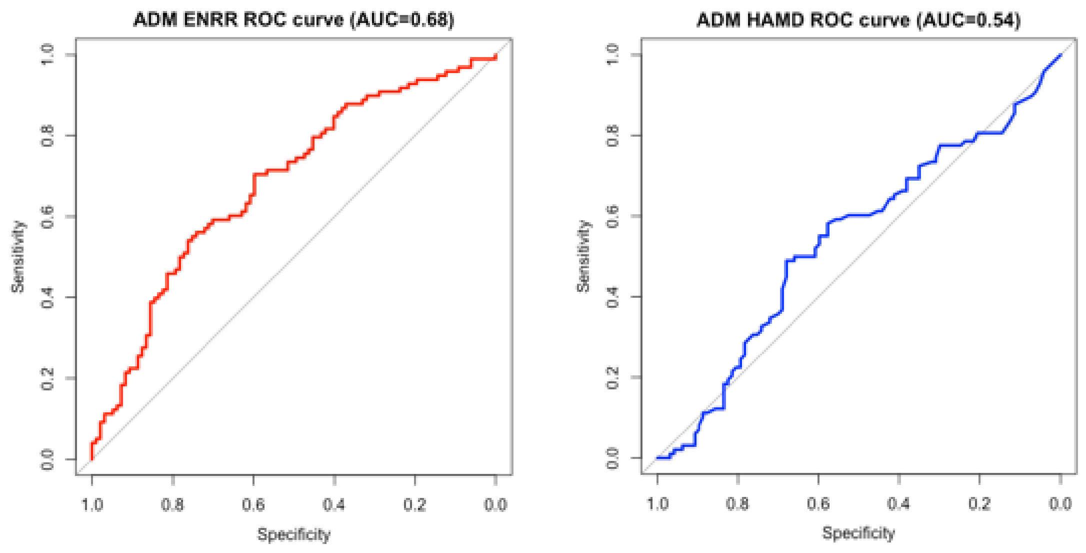

Note. Figure S4 demonstrates the Area Under the Receiver Operating Characteristic (ROC) Curve (AUC), which delineates the relative sensitivity (true positive rate) and specificity (false positive rate) of the prognostic multivariable ADM elastic net (ENRR) model (left, in red) and the baseline comparison ADM Hamilton Depression Scale (HAMD) model (right, in blue). The AUC (red or blue line) is plotted against the straight grey line, which represents the threshold at which the model has no predictive utility. The grey line delineates the likelihood of someone above and below that threshold on the prognostic index has an equal likelihood of relapse. That is, the larger (further away from the grey line) the AUC the greater a model's predictive utility.

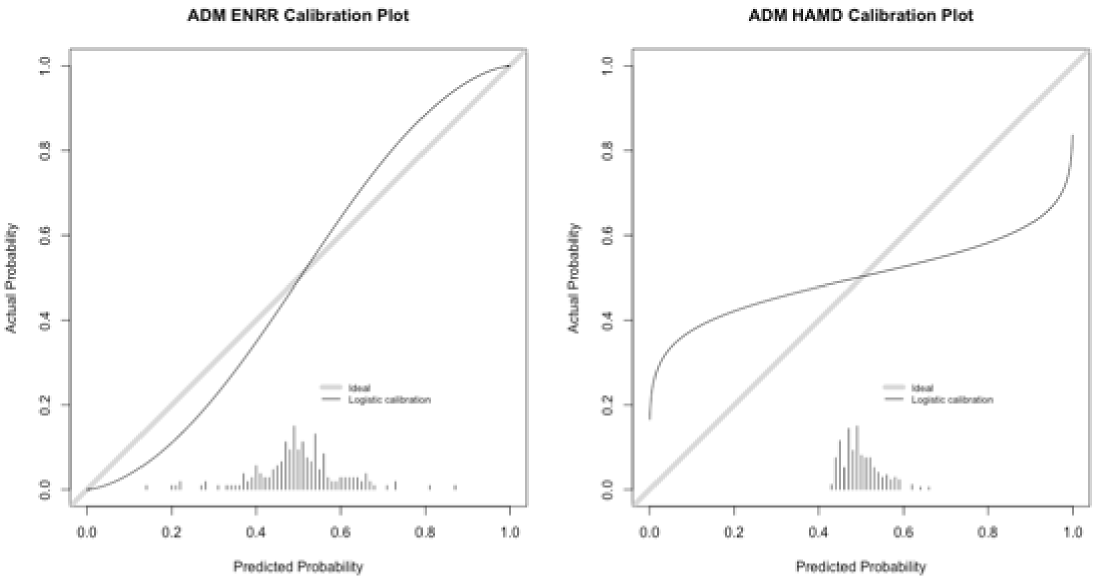

ADM ENRR Calibration Plot: Intercept = –0.02; Slope = 1.49

ADM HAMD Calibration Plot: Intercept = 0.01; Slope = 0.23

We used two tertiles to divide the sample into three groups (based on risk of relapse in ADM) that we labelled: good ADM prognosis, moderate ADM prognosis, and poor ADM prognosis. Sample sizes and descriptive statistics (i.e., means, standard deviations, and ranges) for the ADM prognoses for three groups, broken down by treatment received, are described in Table S3.

Modeling MBCT Prognosis

Using observed depressive relapse (yes/no) over 24 months to evaluate the factual predictions in the MBCT model that had been made without the use of each patient’s own data, the AUC for the MBCT elastic net model was 0.54 (Figure S5). The AUC for the MBCT HAMD comparison model was 0.52. A one-tailed DeLong test failed to reject the null hypothesis that the true difference in AUC between the elastic model and the HAMD model was equal to zero (z=0.37, p=.35), which indicates that the MBCT ENRR model’s

performance was not superior to the MBCT HAMD model. Figure S4 depicts these two ROC curves.

Figure S5

Area Under the Receiver Operating Characteristic (ROC) Curve (AUC) for MBCT Prognostic Models

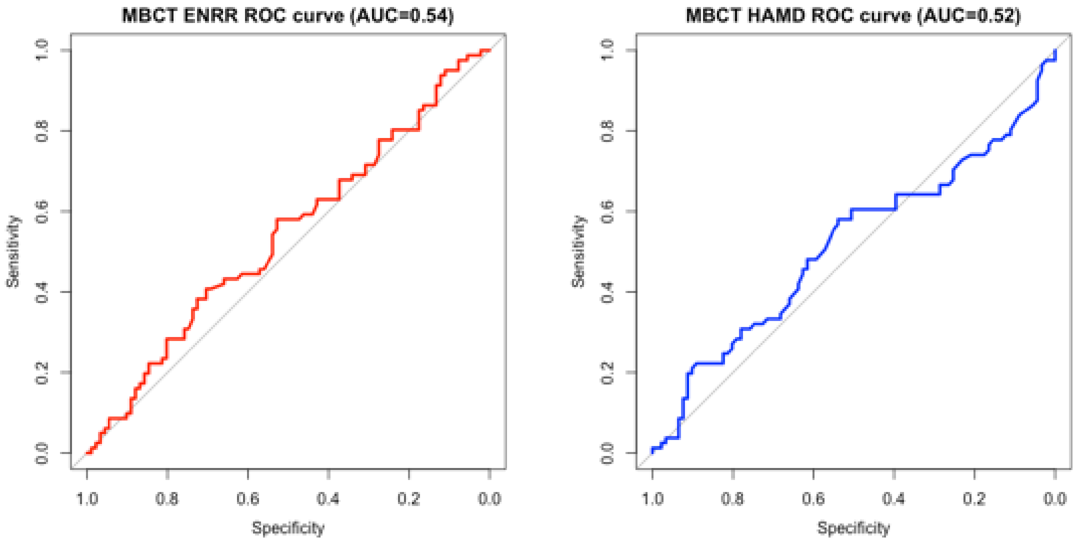

Note. Figure S5 presents the Area Under the Receiver Operating Characteristic (ROC) Curve (AUC), which delineates the relative sensitivity (true positive rate) and specificity (false positive rate) of the prognostic multivariable MBCT elastic net (ENRR) model (left, in red) and the baseline comparison MBCT Hamilton Depression Scale (HAMD) model (right, in blue). The AUC (red or blue line) is plotted against the straight grey line, which represents the threshold at which the model has no predictive utility. The grey line delineates the likelihood of someone above and below that threshold on the prognostic index has an equal likelihood of relapse. That is, the larger (further away from the grey line) the AUC the greater a model's predictive utility.

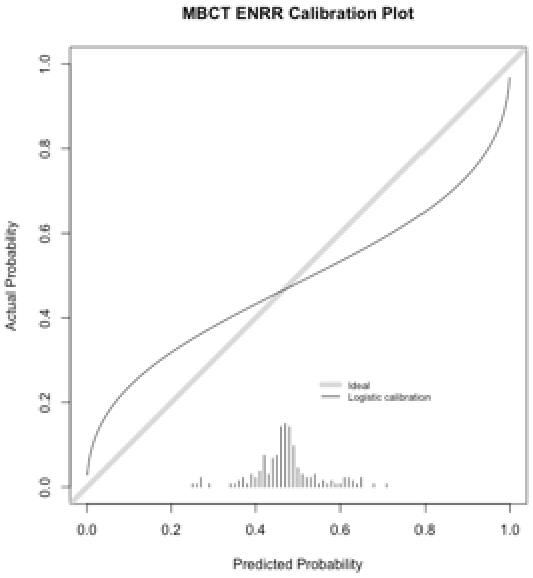

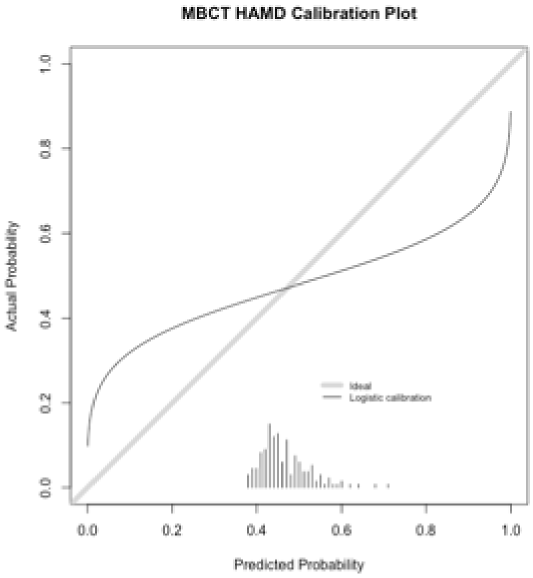

MBCT ENRR Calibration Plot: Intercept = –0.07; Slope = 0.50

MBCT HAMD Calibration Plot: Intercept = –0.08; Slope = –0.31

Table S3

Prognoses from ADM Elastic Net (ENRR) Models Summarized by Subgroups

Prognosis type	*n*	*M*	*SD*	min	max
Good	123	0.404	0.059	0.197	0.473
Good (got ADM)	61	0.407	0.068	0.197	0.472
Good (got MBCT)	62	0.401	0.049	0.294	0.473
Moderate	123	0.501	0.016	0.473	0.530
Moderate (got ADM)	67	0.501	0.016	0.475	0.530
Moderate (got MBCT)	56	0.502	0.015	0.473	0.527
Poor	121	0.591	0.052	0.531	0.772
Poor (got ADM)	67	0.594	0.057	0.531	0.772
Poor (got MBCT)	54	0.589	0.045	0.532	0.703

Note. Good = Participants who have a good prognosis across the 24-month follow-up (i.e., low likelihood of relapse) as indicated by their baseline scores on the variables included in the predictive model; Moderate = Participants who have a moderate prognosis across the 24-month follow-up (i.e., moderate likelihood of relapse) as indicated by their baseline scores on the variables included in the predictive model; Poor = Participants who have a poor prognosis across the 24-month follow-up (i.e., high likelihood of relapse) as indicated by their baseline scores on the variables included in the predictive model; got MBCT = refers to participants who were randomized to the Mindfulness-Based Cognitive Therapy group; got ADM = refers to participants who were randomized to the maintenance of antidepressant medication condition.

Variable Selection Results

Tables S4 and S5 describe, for all 53 variables that were considered, the number of times each variable was retained across the 10 ADM and MBCT (respectively) elastic net models, along with the mean, SD, and range of the associated coefficients. the specific variables that were retained and their associated coefficient weightings varied that were generated.

Table S4

Variable Coefficient Summary for 10-fold Cross-validation of ADM Elastic Net Models

Variable Name	Variable	# times selected	*M*	*SD*	Min	Max
AbuseHL	Level of Parenting Abuse (MOPS)	10	0.34	0.16	0.03	0.57
Chronic	Chronicity	10	-0.33	0.17	-0.60	-0.02
DPES_contentment_pre	DPES Contentment	10	-0.08	0.06	-0.20	-0.01
DPES_joy_pre	DPES Joy	10	-0.05	0.04	-0.12	-0.003
DPES_love_pre	DPES Love	10	-0.07	0.04	-0.14	-0.008
CERTS_negrumin_pre	CERTS Negative Rumination	9	0.05	0.03	0	0.10

CERTS_unresolution_pre	CERTS Unresolution	9	0.07	0.06	0	0.15
CERQ_acceptance_pre	CERQ Acceptance	8	0.04	0.05	0	0.14
Comorbidities	Number of comorbid diagnoses	8	0.03	0.03	0	0.07
FFMQ_actaware_pre	FFMQ Aware	8	-0.04	0.04	-0.11	0
Suicide	Suicide	8	0.10	0.09	0	0.26
BLGSS_TOTAL	Self-Efficacy (GSE)	7	-0.03	0.03	-0.08	0
BLSCIDAgeOnset	Age of depression onset	6	-0.03	0.03	-0.08	0
Age	Age	4	-0.003	0.008	-0.02	0
BDI_TotalB	BDI-II	2	0.001	0.003	0	0.01
Employed	Employed	2	0.01	0.03	0	0.08
SCS_isolation_pre	SCS Isolation	2	-0.01	0.03	-0.11	0
SCS_selfjudge_pre	SCS Self-Judgement	2	-0.01	0.02	-0.05	0
BLSN_TOTAL	Perceived Stigmatisation (SN)	1	0.001	0.002	0	0.01
CERQ_selfblame_pre	CERQ Self-blame	1	0.0002	0.001	0	0.002
CERTS_moveon_pre	CERTS Moving on	1	-0.0001	0.0003	-0.001	0

DPES_awe_pre	DPES Awe	1	-0.0001	0.0004	-0.001	0
DPESb_curiosity_pre	DPES Curiosity	1	0.003	0.01	0	0.03
Fam_hist	Family history of depression	1	0.0001	0.0004	0	0.001
FFMQ_describe_pre	FFMQ Describe	1	-0.0003	0.001	-0.003	0
Gender	Gender	1	0.0005	0.001	0	0.01
Prior_TX	Previous psychological treatment	1	0.004	0.01	0	0.04
SCS_overident_pre	SCS Over-Identification	1	-0.0001	0.0003	-0.001	0
BLRelationships_TOTAL	Relationship Satisfaction (RS)	0	-	-	-	-
BLWS_TOTAL	Recognizing Warning Signs (WS)	0	-	-	-	-
CERQ_catastroph_pre	CERQ Catastrophizing	0	-	-	-	-
CERQ_otherblame_pre	CERQ Other-blame	0	-	-	-	-
CERQ_perspective_pre	CERQ Putting into Perspective	0	-	-	-	-
CERQ_planning_pre	CERQ Refocus on Planning	0	-	-	-	-
CERQ_reapprais_pre	CERQ Positive Reappraisal	0	-	-	-	-
CERQ_refocus_pre	CERQ Positive Refocusing	0	-	-	-	-

CERQ_rumination_pre	CERQ Rumination	0	-	-	-	-
CERTS_constructive_pre	CERTS Constructive Rumination	0	-	-	-	-
CERTS_posrumin_pre	CERTS Positive Rumination	0	-	-	-	-
DPES_compassion_pre	DPES Compassion	0	-	-	-	-
Education	Education	0	-	-	-	-
FFMQ_nonjudge_pre	FFMQ Non-Judging	0	-	-	-	-
FFMQ_nonreact_pre	FFMQ Non-Reactivity	0	-	-	-	-
FFMQ_observe_pre	FFMQ Observe	0	-	-	-	-
HAMD_BL	GRID-HAMD	0	-	-	-	-
PrefADM	Preference for Antidepressant Medication	0	-	-	-	-
PrefCog	Preference for Cognitive Therapy	0	-	-	-	-
PrefWhich	Preference for Therapy Type	0	-	-	-	-
Relationship	Relationship	0	-	-	-	-
SCS_humanity_pre	SCS Common Humanity	0	-	-	-	-

SCS_mindfulness_pre	SCS Mindfulness	0	-	-	-	-
SCS_selfkindness_pre	SCS Self-Kindness	0	-	-	-	-
SCSb_compassionothers_pre	SCS Compassion for others	0	-	-	-	-

Note. Table S4 reports model variable regression coefficient summaries for the ADM prognostic model across the 10-fold cross-validation. Times Selected = Number of times (out of 10) the variable was retained by elastic net regression across the 10-fold cross-validation procedure; Min, Max = minimum, and maximum for variable's coefficient value (includes zeros for when variable was not retained); MOPS = Measure of Parenting Style (Parker et al., 1997); GSE = General Self-Efficacy Scale (Schwarzer et al., 1995); CERQ = Cognitive Emotion Regulation Questionnaire (Garnefski et al., 2001); DPES = Dispositional Positive Emotions Scale (Shiota et al., 2006); CERTS = Cambridge-Exeter Repetitive Thought Scale (Barnard et al., 2007); SCS = Self-Compassion Scale (Neff, 2003); GRID-HAMD = GRID Hamilton Depression Rating Scale (Williams et al., 2008); BDI-II = Beck Depression Inventory Version II (Beck et al., 1996); FFMQ = Five Facet Mindfulness Questionnaire (Baer, 2003). All variables included are described in Table 1.

Table S5

Variable Coefficient Summary for 10-fold Cross-validation of MBCT Elastic Net Models

Variable Name	Variable	# times selected	*M*	*SD*	min	max

SCSb_compassionothers_pre	SCS Compassion for others	10	0.17	0.12	0.05	0.36
Age	Age	7	-0.11	0.11	-0.30	0
DPES_contentment_pre	DPES Contentment	6	-0.06	0.08	-0.22	0
BLSCIDAgeOnset	Age of depression onset	5	-0.01	0.02	-0.05	0
CERTS_posrumin_pre	CERTS Positive Rumination	5	-0.04	0.05	-0.14	0
Suicide	Suicide	5	0.05	0.08	0	0.20
Comorbidities	Number of comorbid diagnoses	4	0.04	0.05	0	0.16
FFMQ_describe_pre	FFMQ Describe	4	0.06	0.08	0	0.17
SCS_humanity_pre	SCS Common Humanity	4	-0.05	0.07	-0.19	0
BLRelationships_TOTAL	Relationship Satisfaction (RS)	3	-0.01	0.01	-0.04	0
HAMD_BL	HAMD	3	0.02	0.05	0	0.13
SCS_isolation_pre	SCS Isolation	3	0.03	0.04	0	0.12
CERQ_planning_pre	CERQ Refocus on Planning	2	0.02	0.03	0	0.08
CERTS_constructive_pre	CERTS Constructive Rumination	2	-0.02	0.04	-0.10	0
Chronic	Chronicity	2	-0.02	0.04	-0.13	0

CERQ_acceptance_pre	CERQ Acceptance	1	0.004	0.01	0	0.04
DPES_joy_pre	DPES Joy	1	-0.002	0.01	-0.02	0
PrefCog	Preference for Cognitive Therapy	1	0.003	0.01	0	0.03
Relationship	Relationship	1	-0.01	0.02	-0.07	0
SCS_mindfulness_pre	SCS Mindfulness	1	-0.004	0.01	-0.04	0
AbuseHL	Level of Parenting Abuse (MOPS)	0	-	-	-	-
BDI_TotalB	BDI-II	0	-	-	-	-
BLGSS_TOTAL	Self-Efficacy (GSE)	0	-	-	-	-
BLSN_TOTAL	Perceived Stigmatisation (SN)	0	-	-	-	-
BLWS_TOTAL	Recognizing Warning Signs (WS)	0	-	-	-	-
CERQ_catastroph_pre	CERQ Catastrophizing	0	-	-	-	-
CERQ_otherblame_pre	CERQ Other-blame	0	-	-	-	-
CERQ_perspective_pre	CERQ Putting into Perspective	0	-	-	-	-
CERQ_reapprais_pre	CERQ Positive Reappraisal	0	-	-	-	-
CERQ_refocus_pre	CERQ Positive Refocusing	0	-	-	-	-

CERQ_rumination_pre	CERQ Rumination	0	-	-	-	-
CERQ_selfblame_pre	CERQ Self-blame	0	-	-	-	-
CERTS_moveon_pre	CERTS Moving on	0	-	-	-	-
CERTS_negrumin_pre	CERTS Negative Rumination	0	-	-	-	-
CERTS_unresolution_pre	CERTS Unresolution	0	-	-	-	-
DPES_awe_pre	DPES Awe	0	-	-	-	-
DPES_compassion_pre	DPES Compassion	0	-	-	-	-
DPES_love_pre	DPES Love	0	-	-	-	-
DPESb_curiosity_pre	DPES Curiosity	0	-	-	-	-
Education	Education	0	-	-	-	-
Employed	Employed	0	-	-	-	-
Fam_hist	Family history of depression	0	-	-	-	-
FFMQ_actaware_pre	FFMQ Aware	0	-	-	-	-
FFMQ_nonjudge_pre	FFMQ Non-Judging	0	-	-	-	-
FFMQ_nonreact_pre	FFMQ Non-Reactivity	0	-	-	-	-

FFMQ_observe_pre	FFMQ Observe	0	-	-	-	-
Gender	Gender	0	-	-	-	-
PrefADM	Preference for Antidepressant Medication	0	-	-	-	-
PrefWhich	Preference for Therapy Type	0	-	-	-	-
Prior_TX	Previous psychological treatment	0	-	-	-	-
SCS_overident_pre	SCS Over-Identification	0	-	-	-	-
SCS_selfjudge_pre	SCS Self-Judgement	0	-	-	-	-
SCS_selfkindness_pre	SCS Self-Kindness	0	-	-	-	-

Note. Table S5 reports model variable regression coefficient summaries for the MBCT (S5) prognostic model across the 10-fold cross-validation. # times selected = Number of times (out of 10) the variable was retained by elastic net regression across the 10-fold cross-validation procedure; Min, Max = minimum, and maximum for variable's coefficient value (includes zeros for when variable was not retained); MOPS = Measure of Parenting Style (Parker et al., 1997); GSE = General Self-Efficacy Scale (Schwarzer et al., 1995); CERQ = Cognitive Emotion Regulation Questionnaire (Garnefski et al., 2001); DPES = Dispositional Positive Emotions Scale (Shiota et al., 2006); CERTS = Cambridge-Exeter Repetitive Thought Scale (Barnard et al., 2007); SCS = Self-Compassion Scale (Neff, 2003); GRID-HAMD = GRID Hamilton Depression

Rating Scale (Williams et al., 2008); BDI-II = Beck Depression Inventory Version II (Beck et al., 1996); FFMQ = Five Facet Mindfulness Questionnaire (Baer, 2003). All variables included are described in Table 1.

SM5 - R Packages

Citations and version information for the software used in data pre-processing, imputation, analyses and visualization are provided below:

- All analyses were performed in **R version 3.5.1**.

R Core Team. (2013). *R: A language and environment for statistical computing*. R Foundation for Statistical Computing. http://www.R-project.org

Packages [version #]

- missForest [1.4]

Stekhoven, D.J. and Buehlmann, P. (2012), *MissForest - nonparametric missing value imputation for mixed-type data*. Bioinformatics, 28(1) 2012, 112-118, doi: 10.1093/bioinformatics/btr597

- glmnet [2.0-16]

Friedman, J., Hastie, T. and Tibshirani, R. (2008) *Regularization Paths for Generalized Linear Models via Coordinate Descent*. Journal of Statistical Software, Vol. 33(1), 1-22 Feb 2010. https://web.stanford.edu/~hastie/Papers/glmnet.pdf and http://www.jstatsoft.org/v33/i01/

- caret [6.0-80]

Kuhn, M. (2008). *Building Predictive Models in R Using the caret Package*. Journal of Statistical Software, 28(5). https://doi.org/10.18637/jss.v028.i05

- pROC [1.13.0]

Xavier Robin, Natacha Turck, Alexandre Hainard, *et al.* (2011) *pROC: an open-source package for R and S+ to analyze and compare ROC curves*. BMC Bioinformatics, **7**, 77. DOI: 10.1186/1471-2105-12-77.

- ggplot2 [3.3.0]

Wickham H (2016). *ggplot2: Elegant Graphics for Data Analysis*. Springer-Verlag New York. ISBN 978-3-319-24277-4, https://ggplot2.tidyverse.org.

- rms [5.1-4]

Harrell, F. E. (2020). *rms: R functions for biostatistical/epidemiologic modeling, testing, estimation, validation, graphics, prediction, and typesetting by storing enhanced model design attributes in the fit*. https://hbiostat.org/R/rms

- survival [3.1-11]

Therneau, T., Grambsch, P., *Modeling Survival Data: Extending the Cox Model*. Springer-Verlag, 2000. https://github.com/therneau/survival

- survminer [0.4.3]

A Kassambara, M Kosinski, & P Biecek. (2017). *survminer: Drawing Survival Curves using'ggplot2'*. https://rpkgs.datanovia.com/survminer/index.html

- RcmdrPlugin.survival [2.5-1]

John Fox, Marilia Sa Carvalho (2012). *The RcmdrPlugin.survival Package: Extending the R Commander Interface to Survival Analysis*. Journal of Statistical Software, 49(7), 1-32.

SM6 - Discussion of Model Components

Several clinical factors were consistently retained in the ADM prognostic models, including as chronicity of depression and history of an abusive childhood, and to a lesser extent disorder comorbidity, history of suicide attempts, and age of depression onset. A number of psychological prediction factors were also consistently retained, and while these psychological factors were initially included in the PREVENT trial as putative predictors of MBCT outcomes, it is useful to consider their role in predicting ADM response. We note, however, that such discussion must always be accompanied by suitable caution concerning interpreting the role of any given *individual* predictor within a multivariable model.

The variable selection-derived finding that history of an abusive childhood was associated with increased risk of relapse in ADM is in line with the original analyses (Kuyken et al., 2015) and previous findings (Williams et al., 2014), which suggests that MBCT may more directly target consequences of high levels of child abuse (e.g., rumination) than ADM (Earley et al., 2014; Kimbrough et al., 2010).

Interestingly greater chronicity of individuals' depression (indexed by the most recent depressive episode lasting for 24 months or more) was associated with lower rates of relapse on ADM. Considering that individuals needed to have remitted on ADM to be included in the PREVENT trial, individuals with a more chronic presentation may have remitted once ADM type and dosage were optimized. Titration of optimal medication dosage and type can be a lengthy process. However, this is purely speculative and

arguably applies just as much individuals whose most recent episode was not classified as chronic.

Higher levels of positive emotions on three of the Dispositional Positive Emotions Scale's subscales (Contentment, Joy, and Love) were associated with lower risk of relapse in ADM. The capacity to experience these positive emotions may be associated with a normalization of abnormal neural responses to positive stimuli in the reward circuitry (Fischer et al., 2021) as well as in the brain circuitry involved in affective processing more generally (Ma, 2015) following ADM treatment.

Elevated scores on the Negative Rumination and Unresolution subscales of the Cambridge-Exeter Repetitive Thought Scale were associated with elevated risk of relapse in 9 of the 10 ADM models. MBCT is known to reduce ruminative thinking (Hölzel et al., 2011; Segal et al., 2013; van der Velden et al., 2015) (though high baseline rumination has been associated with higher dropout from MBCT (Crane & Williams, 2010; Williams et al., 2014)) and post intervention levels of depressive rumination are associated with subsequent relapse (Hölzel et al., 2011; Michalak et al., 2011). Ineffective ruminative thinking (i.e., high on Unresolution) may be insufficiently addressed by pharmacological relapse prevention but instead benefit from the rumination reducing effects of MBCT.

Higher scores on the CERQ-acceptance subscale were associated with greater risk of relapse in 8 of the 10 ADM models, which fits with the psychometric explorations of the CERQ-acceptance subscale in populations with depressive symptoms (Lei et al., 2014; McKinnon et al., 2020). That is, the CERQ-acceptance subscale has been proposed to capture a construct akin to hopelessness in those experiencing recurrent depressive episodes (McKinnon et al., 2020). Although this may appear counter intuitive based on the subscale's name, it has been argued that the subscale (including items such as "cannot change anything about it" and "learn to live with it"), taps into ideas of hopelessness

(Abela, 2001) and arguably learned helplessness (Maier & Seligman, 2016) in depression, rather than content acceptance of the self (McKinnon et al., 2020; Öst, 2014). MBCT is specifically designed to foster acceptance but from a neutral rather than pessimistic perspective and may therefore be particularly benefit individuals with high levels of depressogenic acceptance at baseline.

A higher number of comorbid diagnoses was associated with greater risk of relapse in 8 of 10 ADM models. The finding may be accounted for by the transdiagnostic properties of MBCT. Mindfulness-based interventions have been proposed to target general neurocognitive and affective processes that are shared across disorders (Greeson et al., 2014), such as cognitive flexibility (Shapero et al., 2018; Zou et al., 2020), emotion regulation (Desrosiers et al., 2013; Roemer et al., 2015) and distress tolerance (Brake et al., 2016). In support of its transdiagnostic reach, MBCT has been shown to lead to significant and sustained improvement in mental health problems across a wide range of disorders (Geurts et al., 2021). Consequently individuals who reported higher levels of comorbidity in the PREVENT trial then may have benefited more from being randomized to MBCT versus ADM compared to those with no or fewer comorbid disorders.

Higher scores on the Awareness subscale of the FFMQ at baseline were associated with reduced risk of relapse in 8 of 10 ADM models. Fostering awareness of ones thoughts and feelings is central to mindfulness practice. Individuals who already demonstrated high levels of awareness at baseline may therefore benefit relatively less from mindfulness-based interventions.

A history of attempted suicide was associated with greater risk of relapse in 8 of 10 ADM models. This finding may be accounted for by the benefits that mindfulness-based interventions confer on suicidal ideation and behavior (Forkmann et al., 2014; Williams et al., 2006). Improved distress regulation and reduction of worry have been

proposed as mechanisms through which mindfulness reduces suicidal ideation (Chesin et al., 2016; Forkmann et al., 2014). Moreover, meta-analytic evidence shows that compared to psychological interventions, antidepressant treatment of depression is less effective in reducing suicidal ideation (Boschloo et al., 2019). Together these findings suggest that individuals were at an increased risk of relapse in the ADM condition because MBCT maybe relatively more effective at targeting suicidal ideation compared to ADM.

Higher self-efficacy was associated with reduced risk of relapse in 7 of 10 ADM models. Previous work has shown self-efficacy to be a reliable indicator of individuals' intention to continue ADM treatment. Greater self-efficacy then may have been particularly advantageous in the PREVENT ADM group given the trial's relatively long-term continuation period. Finally, earlier age of depression onset was associated with increased risk of relapse in 6 of 10 ADM models. While the literature on the effectiveness of ADM across age of onset is mixed, the current finding suggests that MBCT may be more effective in targeting more entrenched depression.

SM7 – Table S7. ADM Model from Full ADM Analysis Sample

Variable	Final Model Beta
Intercept	–0.10
Level of Parenting Abuse (MOPS)	0.46
Chronicity	–0.49
DPES Contentment	–0.11
DPES Joy	–0.10
DPES Love	–0.08
CERTS Negative Rumination	0.07
CERTS Unresolution	0.11
CERQ Acceptance	0.10
Number of comorbid diagnoses	0.06
FFMQ Aware	–0.06
Suicide	0.21
Self-Efficacy (GSE)	–0.05
Age of depression onset	–0.05
Age	–0.02

Note. Table S7 reports model variable regression coefficient summaries for an ADM prognostic model constructed via elastic net regression using the full ADM analysis sample (N=195). MOPS = Measure of Parenting Style (Parker et al., 1997); DPES = Dispositional Positive Emotions Scale (Shiota et al., 2006); CERTS = Cambridge-Exeter Repetitive Thought Scale (Barnard et al., 2007); CERQ = Cognitive Emotion Regulation Questionnaire (Garnefski et al., 2001); FFMQ = Five Facet Mindfulness Questionnaire (Baer, 2003); GSE = General Self-Efficacy Scale (Schwarzer et al., 1995). Note, this

model was not used in any analyses for this study, but could be subjected to external validation in future efforts.

Supplementary references

Abela, J. R. (2001). The hopelessness theory of depression: A test of the diathesis–stress and causal mediation components in third and seventh grade children. *Journal of Abnormal Child Psychology*, *29*(3), 241–254.

Baer, R. A. (2003). Mindfulness training as a clinical intervention: A conceptual and empirical review. *Clinical Psychology: Science and Practice*, *10*(2), 125–143.

Barnard, P. J., Watkins, E. R., Mackintosh, B., & Nimmo-Smith, I. (2007). Getting stuck in a mental rut: Some process and experiential attributes. *35th Congress of the British Association for Behavioural and Cognitive Psychothearpies*.

Beck, A. T., Steer, R. A., & Brown, G. K. (1996). *Manual for the Beck Depression Inventory-II*. Psychological Cooperation.

Boschloo, L., Bekhuis, E., Weitz, E. S., Reijnders, M., DeRubeis, R. J., Dimidjian, S., Dunner, D. L., Dunlop, B. W., Hegerl, U., Hollon, S. D., Jarrett, R. B., Kennedy, S. H., Miranda, J., Mohr, D. C., Simons, A. D., Parker, G., Petrak, F., Herpertz, S., Quilty, L. C., … Cuijpers, P. (2019). The symptom-specific efficacy of antidepressant medication vs. cognitive behavioral therapy in the treatment of depression: Results from an individual patient data meta-analysis. *World Psychiatry*, *18*(2), 183–191. https://doi.org/10.1002/wps.20630

Brake, C. A., Sauer-Zavala, S., Boswell, J. F., Gallagher, M. W., Farchione, T. J., & Barlow, D. H. (2016). Mindfulness-Based Exposure Strategies as a Transdiagnostic Mechanism of Change: An Exploratory Alternating Treatment Design. *Behavior Therapy*, *47*(2), 225–238. https://doi.org/10.1016/j.beth.2015.10.008

Chesin, M., Interian, A., Kline, A., Benjamin-Phillips, C., Latorre, M., & Stanley, B. (2016). Reviewing Mindfulness-Based Interventions for Suicidal Behavior. *Archives of Suicide Research*, *20*(4), 507–527. https://doi.org/10.1080/13811118.2016.1162244

Cohen Z. D., Delgadillo J, DeRubeis R. J. (2021) Personalized treatment approaches. In: Barkham M., Lutz W., Castonguay L.G. eds. *Bergin and Garfield's Handbook of Psychotherapy and Behavior Change,* 7th edn. New Jersey: Wiley Blackwell. ISBN: 978-1-119-53658-1

Crane, C., & Williams, J. M. G. (2010). Factors Associated with Attrition from Mindfulness-Based Cognitive Therapy in Patients with a History of Suicidal Depression. *Mindfulness*, *1*(1), 10–20. https://doi.org/10.1007/s12671-010-0003-8

Desrosiers, A., Vine, V., Klemanski, D. H., & Nolen-Hoeksema, S. (2013). Mindfulness and emotion regulation in depression and anxiety: Common and distinct mechanisms of action. *Depression and Anxiety*, *30*(7), 654–661.

Earley, M. D., Chesney, M. A., Frye, J., Greene, P. A., Berman, B., & Kimbrough, E. (2014). Mindfulness intervention for child abuse survivors: A 2.5-year follow-up. *Journal of Clinical Psychology*, *70*(10), 933–941.

Fiedler, K. (2011). Voodoo correlations are everywhere—Not only in neuroscience. *Perspectives on Psychological Science: A Journal of the Association for Psychological Science*, *6*(2), 163–171. https://doi.org/10.1177/1745691611400237

Fischer, A. S., Holt-Gosselin, B., Fleming, S. L., Hack, L. M., Ball, T. M., Schatzberg, A. F., & Williams, L. M. (2021). Intrinsic reward circuit connectivity profiles underlying symptom and quality of life outcomes following antidepressant

medication: A report from the iSPOT-D trial. *Neuropsychopharmacology*, *46*(4), 809–819. https://doi.org/10.1038/s41386-020-00905-3

Forkmann, T., Wichers, M., Geschwind, N., Peeters, F., van Os, J., Mainz, V., & Collip, D. (2014). Effects of mindfulness-based cognitive therapy on self-reported suicidal ideation: Results from a randomised controlled trial in patients with residual depressive symptoms. *Comprehensive Psychiatry*, *55*(8), 1883–1890. https://doi.org/10.1016/j.comppsych.2014.08.043

Garnefski, N., Kraaij, V., & Spinhoven, P. (2001). Negative life events, cognitive emotion regulation and emotional problems. *Personality and Individual Differences*, *30*(8), 1311–1327.

Geurts, D. E. M., Haegens, N. M., Van Beek, M. H. C. T., Schroevers, M. J., Compen, F. R., & Speckens, A. E. M. (2021). Putting mindfulness-based cognitive therapy to the test in routine clinical practice: A transdiagnostic panacea or a disorder specific intervention? *Journal of Psychiatric Research*, *142*, 144–152. https://doi.org/10.1016/j.jpsychires.2021.07.043

Greeson, J., Garland, E. L., & Black, D. (2014). Mindfulness: A transtherapeutic approach for transdiagnostic mental processes. In *The Wiley Blackwell Handbook of Mindfulness: Vol. I and II* (pp. 533–562). Wiley Blackwell. https://doi.org/10.1002/9781118294895.ch28

Hamilton, M. (1967). Development of a rating scale for primary depressive illness. *British Journal of Social and Clinical Psychology*, *6*(4), 278–296.

Hastie, T., Tibshirani, R., & Friedman, J. (2009). *The elements of statistical learning* (2nd ed.). Springer.

Hölzel, B. K., Lazar, S. W., Gard, T., Schuman-Olivier, Z., Vago, D. R., & Ott, U. (2011). How does mindfulness meditation work? Proposing mechanisms of action from a conceptual and neural perspective. *Perspectives on Psychological Science*, *6*(6), 537–559.

Kimbrough, E., Magyari, T., Langenberg, P., Chesney, M., & Berman, B. (2010). Mindfulness intervention for child abuse survivors. *Journal of Clinical Psychology*, *66*(1), 17–33.

Kuyken, W., Hayes, R., Barrett, B., Byng, R., Dalgleish, T., Kessler, D., Lewis, G., Watkins, E., Brejcha, C., Cardy, J., Causley, A., Cowderoy, S., Evans, A., Gradinger, F., Kaur, S., Lanham, P., Morant, N., Richards, J., Shah, P., … Byford, S. (2015). Effectiveness and cost-effectiveness of mindfulness-based cognitive therapy compared with maintenance antidepressant treatment in the prevention of depressive relapse or recurrence (PREVENT): A randomised controlled trial. *Lancet*, *386*(9988), 63–73. https://doi.org/10.1016/S0140-6736(14)62222-4

Lei, H., Zhang, X., Cai, L., Wang, Y., Bai, M., & Zhu, X. (2014). Cognitive emotion regulation strategies in outpatients with major depressive disorder. *Psychiatry Research*, *218*(1), 87–92. https://doi.org/10.1016/j.psychres.2014.04.025

Lorenzo-Luaces, L., Peipert, A., De Jesús Romero, R., Rutter, L. A., & Rodriguez-Quintana, N. (2020). Personalized Medicine and Cognitive Behavioral Therapies for Depression: Small Effects, Big Problems, and Bigger Data. *International Journal of Cognitive Therapy*. https://doi.org/https://doi.org/10.1007/s41811-020-00094-3

Ma, Y. (2015). Neuropsychological mechanism underlying antidepressant effect: A systematic meta-analysis. *Molecular Psychiatry*, *20*(3), 311–319. https://doi.org/10.1038/mp.2014.24

Maier, S. F., & Seligman, M. E. (2016). Learned helplessness at fifty: Insights from neuroscience. *Psychological Review*, *123*(4), 349.

McKinnon, A., Kuyken, W., Hayes, R., Werner-Seidler, A., Watson, P., Dalgleish, T., & Schweizer, S. (2020). The psychometric properties of the cognitive emotion regulation questionnaire (CERQ) in a clinical sample of adults with recurrent depression. *Journal of Affective Disorders*, *276*, 212–219. https://doi.org/10.1016/j.jad.2020.06.061

Michalak, J., Hölz, A., & Teismann, T. (2011). Rumination as a predictor of relapse in mindfulness-based cognitive therapy for depression. *Psychology and Psychotherapy: Theory, Research and Practice*, *84*(2), 230–236.

Neff, K. D. (2003). The development and validation of a scale to measure self-compassion. *Self and Identity*, *2*(3), 223–250. https://doi.org/10.1080/15298860309027

Olsson-Collentine, A., van Assen, M., & Hartgerink, C. H. J. (2019). The Prevalence of Marginally Significant Results in Psychology Over Time. *Psychol Sci*, *30*(4), 576-586. https://doi.org/10.1177/0956797619830326

Öst, L.-G. (2014). The efficacy of Acceptance and Commitment Therapy: An updated systematic review and meta-analysis. *Behaviour Research and Therapy*, *61*, 105–121.

Parker, G., Roussos, J., Hadzi-Pavlovic, D., Mitchell, P., Wilhelm, K., & Austin, M. P. (1997). The development of a refined measure of dysfunctional parenting and assessment of its relevance in patients with affective disorders. *Psychological Medicine*, *27*(5), 1193–1203.

Riley, R. D., Snell, K. I. E., Martin, G. P., Whittle, R., Archer, L., Sperrin, M., & Collins, G. S. (2021). Penalization and shrinkage methods produced unreliable clinical prediction models especially when sample size was small. *Journal of Clinical Epidemiology*, *132*, 88-96. https://doi.org/10.1016/j.jclinepi.2020.12.005

Roemer, L., Williston, S. K., & Rollins, L. G. (2015). Mindfulness and emotion regulation. *Current Opinion in Psychology*, *3*, 52–57. https://doi.org/10.1016/j.copsyc.2015.02.006

Schwarzer, R., Jerusalem, M., Wright, S., & Johnston, M. (1995). Generalized self-efficacy scale. In *Measures in health psychology: A user's portfolio* (pp. 35–37). NFER-Nelson.

Segal, Z. V., Williams, J. M. G., & Teasdale, J. D. (2013). *Mindfulness-Based Cognitive Therapy for Depression, Second Edition* (2nd ed.). Guilford Press.

Shapero, B. G., Greenberg, J., Mischoulon, D., Pedrelli, P., Meade, K., & Lazar, S. W. (2018). Mindfulness-Based Cognitive Therapy Improves Cognitive Functioning and Flexibility Among Individuals with Elevated Depressive Symptoms. *Mindfulness*, *9*(5), 1457–1469. https://doi.org/10.1007/s12671-018-0889-0

Shiota, M. N., Keltner, D., & John, O. P. (2006). Positive emotion dispositions differentially associated with Big Five personality and attachment style. *CH09.Indd 345 10/5/09 7:17:15 PM Emotion*.

van der Velden, A. M., Kuyken, W., Wattar, U., Crane, C., Pallesen, K. J., Dahlgaard, J., Fjorback, L. O., & Piet, J. (2015). A systematic review of mechanisms of change in mindfulness-based cognitive therapy in the treatment of recurrent major depressive disorder. *Clinical Psychology Review*, *37*, 26–39.

Williams, J. B. W., Kobak, K. A., Bech, P., Engelhardt, N., Evans, K., Lipsitz, J., Olin, J., Pearson, J., & Kalali, A. (2008). The GRID-HAMD: Standardization of the Hamilton depression rating scale. *International Clinical Psychopharmacology*, *23*(3), 120–129.

Williams, J. M. G., Crane, C., Barnhofer, T., Brennan, K., Duggan, D. S., Fennell, M. J. V., Hackmann, A., Krusche, A., Muse, K., & Von Rohr, I. R. (2014).

Mindfulness-based cognitive therapy for preventing relapse in recurrent depression: A randomized dismantling trial. *Journal of Consulting and Clinical Psychology*, *82*(2), 275–286.

Williams, J. M. G., Duggan, D. S., Crane, C., & Fennell, M. J. V. (2006). Mindfulness-Based cognitive therapy for prevention of recurrence of suicidal behavior. *Journal of Clinical Psychology*, *62*(2), 201–210. https://doi.org/10.1002/jclp.20223

Zou, Y., Li, P., Hofmann, S. G., & Liu, X. (2020). The Mediating Role of Non-reactivity to Mindfulness Training and Cognitive Flexibility: A Randomized Controlled Trial. *Frontiers in Psychology*, *0*. https://doi.org/10.3389/fpsyg.2020.01053

TRIPOD Checklist: Prediction Model Development and Validation

Section/Topic	Item		Checklist Item	Page
**Title and abstract**				
Title	1	D;V	Identify the study as developing and/or validating a multivariable prediction model, the target population, and the outcome to be predicted.	1
Abstract	2	D;V	Provide a summary of objectives, study design, setting, participants, sample size, predictors, outcome, statistical analysis, results, and conclusions.	4
**Introduction**				
Background and objectives	3a	D;V	Explain the medical context (including whether diagnostic or prognostic) and rationale for developing or validating the multivariable prediction model, including references to existing models.	7
	3b	D;V	Specify the objectives, including whether the study describes the development or validation of the model or both.	8
**Methods**				
Source of data	4a	D;V	Describe the study design or source of data (e.g., randomized trial, cohort, or registry data), separately for the development and validation data sets, if applicable.	8
	4b	D;V	Specify the key study dates, including start of accrual; end of accrual; and, if applicable, end of follow-up.	
Participants	5a	D;V	Specify key elements of the study setting (e.g., primary care, secondary care, general population) including number and location of centres.	6
	5b	D;V	Describe eligibility criteria for participants.	6
	5c	D;V	Give details of treatments received, if relevant.	6
Outcome	6a	D;V	Clearly define the outcome that is predicted by the prediction model, including how and when assessed.	20
	6b	D;V	Report any actions to blind assessment of the outcome to be predicted.	
Predictors	7a	D;V	Clearly define all predictors used in developing or validating the multivariable prediction model, including how and when they were measured.	Table 1
	7b	D;V	Report any actions to blind assessment of predictors for the outcome and other predictors.	
Sample size	8	D;V	Explain how the study size was arrived at.	8, Figure S1
Missing data	9	D;V	Describe how missing data were handled (e.g., complete-case analysis, single imputation, multiple imputation) with details of any imputation method.	16
Statistical analysis methods	10a	D	Describe how predictors were handled in the analyses.	16, Figure 1
	10b	D	Specify type of model, all model-building procedures (including any predictor selection), and method for internal validation.	17-20, 23, Figure 1
	10c	V	For validation, describe how the predictions were calculated.	
	10d	D;V	Specify all measures used to assess model performance and, if relevant, to compare multiple models.	24-25
	10e	V	Describe any model updating (e.g., recalibration) arising from the validation, if done.	
Risk groups	11	D;V	Provide details on how risk groups were created, if done.	25
Development vs. validation	12	V	For validation, identify any differences from the development data in setting, eligibility criteria, outcome, and predictors.	
**Results**				
Participants	13a	D;V	Describe the flow of participants through the study, including the number of participants with and without the outcome and, if applicable, a summary of the follow-up time. A diagram may be helpful.	Figure S1
	13b	D;V	Describe the characteristics of the participants (basic demographics, clinical features, available predictors), including the number of participants with missing data for predictors and outcome.	Tables S1 & S2
	13c	V	For validation, show a comparison with the development data of the distribution of important variables (demographics, predictors and outcome).	
Model development	14a	D	Specify the number of participants and outcome events in each analysis.	throughout
	14b	D	If done, report the unadjusted association between each candidate predictor and outcome.	
Model specification	15a	D	Present the full prediction model to allow predictions for individuals (i.e., all regression coefficients, and model intercept or baseline survival at a given time point).	Table S7
	15b	D	Explain how to the use the prediction model.	
Model performance	16	D;V	Report performance measures (with CIs) for the prediction model.	25-26, 28-29
Model-updating	17	V	If done, report the results from any model updating (i.e., model specification, model performance).	
**Discussion**				
Limitations	18	D;V	Discuss any limitations of the study (such as nonrepresentative sample, few events per predictor, missing data).	32-34
Interpretation	19a	V	For validation, discuss the results with reference to performance in the development data, and any other validation data.	
	19b	D;V	Give an overall interpretation of the results, considering objectives, limitations, results from similar studies, and other relevant evidence.	31-34
Implications	20	D;V	Discuss the potential clinical use of the model and implications for future research.	34-35
**Other information**				
Supplementary information	21	D;V	Provide information about the availability of supplementary resources, such as study protocol, Web calculator, and data sets.	8
Funding	22	D;V	Give the source of funding and the role of the funders for the present study.	2-3

TRIPOD Checklist: Prediction Model Development and Validation

*Items relevant only to the development of a prediction model are denoted by D, items relating solely to a validation of a prediction model are denoted by V, and items relating to both are denoted D;V. We recommend using the TRIPOD Checklist in conjunction with the TRIPOD Explanation and Elaboration document.
